# scQA: A dual-perspective cell type identification model for single cell transcriptome data

**DOI:** 10.1016/j.csbj.2023.12.021

**Published:** 2023-12-21

**Authors:** Di Li, Qinglin Mei, Guojun Li

**Affiliations:** a Research Center for Mathematics and Interdisciplinary Sciences, Shandong University, Qingdao 266237, China; b MOE Key Laboratory of Bioinformatics, BNRIST Bioinformatics Division, Department of Automation, Tsinghua University, Beijing 100084, China

**Keywords:** Single-cell RNA-seq, Dropout, Feature extraction, Label propagation, Bidirectional clustering

## Abstract

Single-cell RNA sequencing technologies have been pivotal in advancing the development of algorithms for clustering heterogeneous cell populations. Existing methods for utilizing scRNA-seq data to identify cell types tend to neglect the beneficial impact of dropout events and perform clustering focusing solely on quantitative perspective. Here, we introduce a novel method named scQA, notable for its ability to concurrently identify cell types and cell type-specific key genes from both qualitative and quantitative perspectives. In contrast to other methods, scQA not only identifies cell types but also extracts key genes associated with these cell types, enabling bidirectional clustering for scRNA-seq data. Through an iterative process, our approach aims to minimize the number of landmarks to approximately a dozen while maximizing the inclusion of quasi-trend-preserved genes with dropouts both qualitatively and quantitatively. It then clusters cells by employing an ingenious label propagation strategy, obviating the requirement for a predetermined number of cell types. Validated on 20 publicly available scRNA-seq datasets, scQA consistently outperforms other salient tools. Furthermore, we confirm the effectiveness and potential biological significance of the identified key genes through both external and internal validation. In conclusion, scQA emerges as a valuable tool for investigating cell heterogeneity due to its distinctive fusion of qualitative and quantitative facets, along with bidirectional clustering capabilities. Furthermore, it can be seamlessly integrated into border scRNA-seq analyses. The source codes are publicly available at https://github.com/LD-Lyndee/scQA.

## Graphical Abstract

**Figure:**
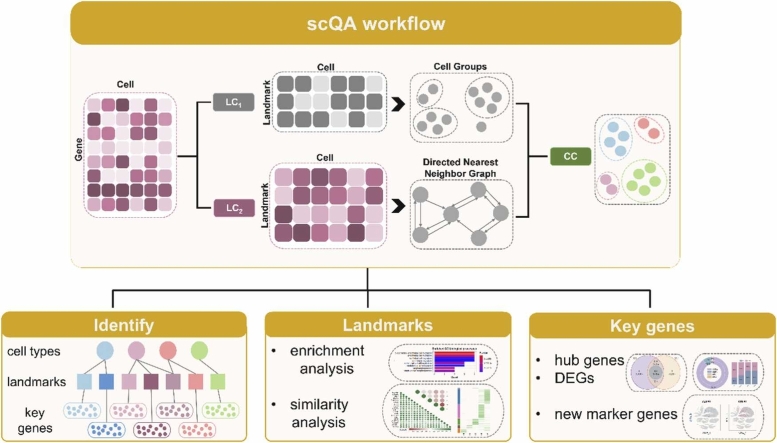

## 1 Introduction

Advances in single cell RNA sequencing (scRNA-seq) technologies have revolutionized the study of development and cellular heterogeneity of transcriptomes at single-cell resolution and enhanced to understand how cells contribute to complex and dynamic systems [1], [2]. As sequencing costs decrease, there has been a substantial rise in the production of scRNA-seq data varying in scale and accuracy [3], [4], [5].

A prevalent occurrence observed in scRNA-seq data across different protocols is known as “dropout”, describing zero expression for genes that show non-zero expression in only a proportion of cells [6]. These dropout events can be attributed to various factors, spanning both technical anomalies and biological influences [7]. Over time, researchers have devised multiple approaches to tackle the challenge of high sparseness caused by dropouts. One commonly employed technique is dimensionality reduction, specifically principal component analysis (PCA) [8]. For instance, SC3 (single-cell consensus clustering) [9] is a consensus method applying *k*-means on transformed distance matrices using PCA or eigenvectors of Laplacian, and ultimately classifies cells through hierarchical clustering. After performing PCA, RCSL [10] considers both local similarity and global similarity for cell clustering. Seurat [11] leverages shared nearest neighbor graphs and Louvain community detection following PCA. Another popular method, CIDR (clustering through imputation and dimensionality reduction) [12] employs PCA while considering dropout events to improve similarities. Nowadays, the integration of deep learning into scRNA-seq data analysis is advancing rapidly, such as scGNN [13], [14], which incorporates graph convolutional network into multi-modal autoencoders for gene imputation and cell clustering. Beyond employing dimensionality reduction approaches to alleviate the impact of high dimensionality and sparsity, there are specialized imputation methods to substitute zeros with numerical values by fitting data with a moderate model, such as MAGIC [15] and ScImpute [16].

Recently, there has been a shift in perspective regarding dropouts, underscored by the literatures indicating that dropouts may possess inherent value. Qiu [17] clusters cells based solely on binary information, yielding results consistent with other methods. M3Drop [18] capitalizes on genes with elevated dropout rates as valuable attributes for downstream analyses. scBFA [19] demonstrates the efficacy of features derived from binary PCA to achieve precise cell clustering. Amid the prevalent perspective of considering dropouts as noise, Kim [20] emphasizes that implementing imputation or normalization prior to the cellular heterogeneity may lead to inevitable loss of biological signals in unique molecular identifiers-based (UMI-based) data. Simultaneously, ample evidence indicates a reduction in technical zeros within UMI-based data, and modeling zeros using different methods may lead to considerable discrepancies for downstream analyses [21], [22], [23]. Consequently, selecting an approach to handle zeros should be cautious, as simplistically treating dropouts as noise may distort the original data.

Inspired by what mentioned above, we propose a new method called scQA (a model for clustering **S**ingle-**C**ell RNA-seq data based on **Q**ualitative and **Q**uantitative **A**nalysis), which can effectively and efficiently cluster cells across diverse scales from dual perspectives based on so called landmarks which indicate the consensus expression vectors of the genes with similar expression patterns. From the qualitative perspective, we regard dropouts as weak signals, strengthened by amalgamation of genes sharing analogous binary expression patterns and performing consensus, yielding a binary low-dimensional representation of cells. From the quantitative perspective, we introduce the concept of quasi-trend-preserved genes, referring to genes that exhibit similar trends of expression, that is, the ranks defined by expression vectors are akin, although they might not be identical [24] (Supplementary Note S1), and consistently cluster genes exhibiting quasi-trend-preserved patterns to derive the quantitative low-dimensional representation of cells. To the best of our knowledge, this is the first method that integrates both perspectives for cell type identification, while other methods typically focus on either the quantitative or qualitative perspective exclusively. Subsequently, cells are clustered via an ingenious label propagation strategy combining both two sources of features. We initially establish the core of clusters by generating seeds, followed by propagating labels along both the outgoing and incoming edges of a directed graph with a fixed path length. To improve precision, we implement a mechanism designed to prevent misclassification, ultimately resulting in precise cell classification along with the identification of key genes associated with each cluster, thereby achieving bidirectional clustering. Tested on 20 public scRNA-seq datasets with well-annotated cell types, scQA substantially outperforms other notable tools while maintaining efficiency. Furthermore, we demonstrate that scQA captures key genes sharing attributes with differentially expressed genes, hub genes and marker genes, serving as reliable landmarks for clustering cells and possessing the capability to detect new marker genes.

## 2 Methods

### 2.1 Overview of scQA

**Fig. 1:**
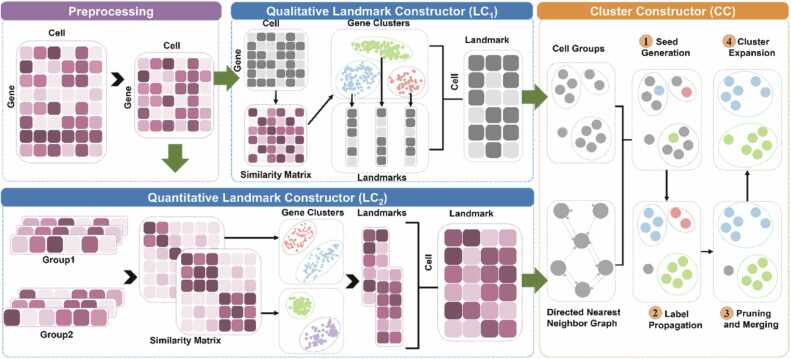
Illustration of scQA. The raw gene expression matrix is processed by filtration, log-transformed and highly variable genes are retained. Qualitative landmark constructor (LC_1_) initially binarizes the matrix and persists to cluster genes with qualitative quasi-trend-preserved expression patterns to form landmarks. Quantitative landmark constructor (LC_2_) divides genes into several groups and creates landmarks by clustering genes within each group, resulting in clusters of quantitative quasi-trend-preserved genes. In cluster constructor (CC) module, a directed *k*-nearest neighbor graph is constructed using the matrix generated by LC_2_ and cells are grouped based on the matrix generated by LC_1_. For each group, vertices with high scores are identified as seed candidates. Each seed is assigned a unique label, which propagates to form a cluster. Then, clusters are scrutinized and merged to obtain highly reliable clusters. All unlabeled vertices are either added to existing clusters or isolated to create new ones in the cluster expansion step.

The scQA framework comprises three primary modules: qualitative landmark constructor (LC_1_), quantitative landmark constructor (LC_2_), and cluster constructor (CC). LC_1_ and LC_2_ focus on feature extraction by generating the consensus expression pattern for every cluster of quasi-trend-preserved genes, known as a landmark. The third module aims to cluster cells through an innovative label propagation strategy. The pipeline of scQA is depicted in Fig. 1.

### 2.2 Data pre-processing

To address sparsity in raw scRNA-seq data, we initially filter out genes expressed in fewer than x% cells or more than (100−x)% cells, with x set to 2.5. To identify genes containing valuable information, we retain 2000 highly variable genes, employing the method outlined in references [11], [25]. All data are log-transformed and normalized to ensure that each entry falls in the range [0,1]. The resulting preprocessed gene expression matrix, denoted as $D_{m\times n}$, encompasses m= 2000 informative genes and n cells. In D, the element $d_{\mathrm{ij}}$ signifies the expression value of gene i in cell j.

### 2.3 Qualitative landmark constructor

The LC_1_ module is responsible for generating landmarks to create a binary low-dimensional representation. The pseudocode for this process is depicted in Agorithm1.

For each gene in D, we define a binary vector as:

(1) $$\begin{array}{c} G_{i}=\left(g_{i1},g_{i2},\ldots ,g_{\mathrm{in}}\right),g_{\mathrm{ij}}=\left\{\begin{array}{c} 0,\&d_{\mathrm{ij}}=0\\ 1,\&d_{\mathrm{ij}}>0 \end{array}\right) \end{array}\;$$

To enhance the computational efficiency and robustness of clustering, genes are initially sorted based on the decreasing number of zeros in vectors. For $G_{i}$, we take 0.05×m genes before and after $G_{i}$ in the sorted sequence as the neighbors of $G_{i}$, and compute the similarity between $G_{i}$ and each neighbor $G_{j}$ as:

(2) $$\begin{array}{c} S\left(G_{i},G_{j}\right)=1-\frac{1}{n}h\left(G_{i},G_{j}\right),h\left(G_{i},G_{j}\right)=\sum_{k=1}^{n}\left|g_{\mathrm{ik}}-g_{\mathrm{jk}}\right| \end{array}$$

We construct a gene similarity graph by representing genes as vertices and top 1999 gene pairs (one thousandth of all gene pairs) based on similarity scores as edges. The initial gene clusters are formed by connected components in the graph that contain more than two vertices. We define a template vector$\;T_{r}={(t}_{r1},t_{r2},\cdots t_{\mathrm{rn}})$ for each cluster $C_{r}$, where each term is defined as:

(3) $$\begin{array}{c} t_{\mathrm{rk}}=\left\{\begin{array}{c} 1,\&\mathrm{if}\;\sum_{G_{j}\in C_{r}}\frac{g_{\mathrm{jk}}}{\left|C_{r}\right|}\geq 0.5\\ 0,\&\mathrm{otherwise} \end{array}\right) \end{array}$$

To assign genes outside clusters, we devise a method to accurately and reliably determine the similarity score between a gene $G_{i}$ and a cluster $C_{j}$. By $\mu_{\mathrm{ijk}}$ we denote the similarity increment contributed by $G_{i}$ and $C_{j}$ at the *k*th component:

(4) $$\begin{array}{c} \mu_{\mathrm{ijk}}=\left\{\begin{array}{c} 1,\&g_{\mathrm{ik}}=t_{\mathrm{jk}}\\ 0,\&g_{\mathrm{ik}}=1\mathrm{and}\;t_{\mathrm{jk}}=0\\ a_{\mathrm{jk}},\&g_{\mathrm{ik}}=0\mathrm{and}\;t_{\mathrm{jk}}=1 \end{array}\right) \end{array}$$

where $a_{\mathrm{jk}}$ is *k*th component of a subsidiary vector $A_{j}$ defined for each cluster $C_{j}$ as follows:

(5) $$\begin{array}{c} a_{\mathrm{jk}}=\max\left(0.5-\frac{\sum_{G_{p}\in C_{j}}d_{\mathrm{pk}}}{\left|C_{j}\right|},0\right) \end{array}$$

The similarity ${S(G}_{i},C_{j})$ between $G_{i}$ and $C_{j}$ is defined as average similarity increments contributed by $G_{i}$ and $C_{j}$ at all components:

(6) $$\begin{array}{c} S\left(G_{i},C_{j}\right)=\frac{1}{n}\sum_{k=1}^{n}\mu_{\mathrm{ijk}} \end{array}$$

The rationality can be strongly inferred from the definition of similarity alone: the *k*th component contributes a maximum score of 1 to the overall similarity when$\;g_{\mathrm{ik}}=t_{\mathrm{jk}}$; the *k*th component contributes a null score when genes in $C_{j}$ do not express at the *k*th component while $G_{i}$ does. For the last situation, notice that $g_{\mathrm{ik}}=0$ could occur when the expression value of $G_{i}$ under *k*th cell was not observed due to its weak signal. In other words, there is a possibility that $G_{i}$ may have a positive expression value, albeit not prominently, even when $g_{\mathrm{ik}}=0$, and, the likelihood decreases as the expression becomes more prominent as defined in (5).

We iteratively calculate the similarity ${S(G}_{i},C_{j})$. $G_{i}$ is added to $C_{J}$ if ${S(G}_{i},C_{J})$ exceeds a prespecified parameter ρ, and we update $T_{J}$ and $A_{J}$ as:

(7) $$\begin{array}{c} t_{\mathrm{Jk}}=\left\{\begin{array}{c} t_{\mathrm{Jk}},\&\mathrm{if}\;g_{\mathrm{ik}}=t_{\mathrm{Jk}}\\ \left[\frac{\sum_{G_{p}\in C_{J}}g_{\mathrm{pk}}}{\left|C_{J}\right|}\right],\&\mathrm{if}\;g_{\mathrm{ik}}\neq t_{\mathrm{Jk}} \end{array}\right),a_{\mathrm{Jk}}=\max\left(0.5-\frac{\sum_{G_{p}\in C_{J}}d_{\mathrm{pk}}}{\left|C_{J}\right|},0\right) \end{array}$$

Otherwise, $G_{i}$ forms a new singleton cluster (Algorithm 1, lines:13–15). After clustering all genes, we remove clusters with less than 3 genes, and reduce the parameter ρ for another round iteration if necessary. By default, we perform one iteration with the fixed parameter ρ set to 0.7. Then we utilize all retained clusters to create a qualitative cell-landmark matrix $Q_{1}$ with cells corresponding to rows and landmarks to columns. Each column represents the consensus pattern of its respective cluster (Algorithm 1, line:17).

Algorithm 1 Construct Qualitative Landmarks.

**Table:** 

### 2.4 Quantitative landmark constructor

The LC_2_ module is strategically employed to form the quantitative low-dimensional representation of cells with the pseudocode being presented in Algorithm 2.

We first discretize the continuous expression values by binning, which entails dividing the interval$\;\left[0,1\right]\;$into k bins:

(8) $$\begin{array}{c} \left[0,0\right],\left(0,\left(1/\left(k-1\right)\right]\right),\left(1/\left(k-1\right)\right),\left(2/\left(k-1\right)\right],\ldots ,\left(\left(k-2\right)/\left(k-1\right)\right),\left(1\right] \end{array}$$

The default value of k is set to 6 in our experiments. The matrix D can be qualitatively represented as D′, where $d_{\mathrm{ij}}$ is replaced by the bin index s if and only if $d_{\mathrm{ij}}$ falls in sth bin, then genes are grouped based on the number of bins they utilize.

For each group of genes, we derive the initial clusters following the same approach as LC_1_ by calculating similarity scores using Spearman's rank correlation coefficient. The template vector$\;T_{r}$ for cluster $C_{r}$ is defined as:

(9) $$\begin{array}{c} t_{\mathrm{rk}}=\sum_{G_{j}\in C_{r}}\frac{d_{\mathrm{jk}}}{\left|C_{r}\right|} \end{array}$$

For clustering genes of quasi-trend-preserved, we define upper and lower bound vectors ${\mathrm{TU}}_{j}$ and ${\mathrm{TL}}_{j}$ for each cluster $C_{j}$. The lower (resp. upper) bound vector ${\mathrm{TL}}_{j}$ (resp. ${\mathrm{TU}}_{j}$) with its kth component being the element at the lower (resp. upper) quartile $x\%|C_{j}|$ (resp. $(1-x)\%|C_{j}|$) in an ordered sequence of $\left|C_{j}\right|$ elements in the kth component of cluster $C_{j}$.

For each $G_{i}$ outside clusters, we calculate the similarity ${S(G}_{i},C_{j})$ as the ratio between the number of components where the expression values of $G_{i}$ fall between ${\mathrm{TL}}_{j}$ and ${\mathrm{TU}}_{j}$ of $C_{j}$ and n. Based on ${S(G}_{i},C_{j})$, we iteratively cluster gene $G_{i}$ and prune clusters in the same fashion as we did in LC_1_ (Algorithm 2, lines:9–21). Finally, all retained clusters of all groups are jointed to create a single quantitative cell-landmark matrix $Q_{2}$ with cells corresponding to rows and landmarks to columns representing consensus of clusters (Algorithm 2, line:22).

Algorithm 2 Construct Quantitative Landmarks.

**Table:** 

### 2.5 Cluster constructor

CC module clusters cells by combination of the two cell-landmark matrices derived from LC_1_ and LC_2_ modules, as outlined in the pseudocode of Algorithm 3. We construct a directed nearest neighbor graph G with nodes representing cells, and edges representing cell pairs with higher similarity measured using Pearson correlation coefficient based on $Q_{2}$ to avoid bias from using the same similarity metric as in LC_2_. For each node in G, it has 0.1 *n* (resp. 0.05 *n*) out neighbors if n<10,000 (resp. n≥10,000). The CC module is implemented in four steps:

#### 2.5.1 Seed generation

We first group cells based on $Q_{1}$, ensuring that cells in the same group share identical row vector in $Q_{1}$. One of ideas behind scQA is that cells within the same group should comprise the core of the final cluster to be tested. In light of this, we can generate all potential seeds with the guidance of the core groups. Doing so, we assign to each node a density d, calculated as the average of similarities between the node and all its neighbors. Intuitively, nodes with higher density are more likely to be authentic seeds. We determine a threshold β that a node of its density beyond should be qualified to be a reliable seed. This is achieved by recording the maximum density within each group, and the minimum one among the maximum densities across groups is used as the threshold β. If two seed candidates that are mutually neighboring in G, we merge them into a single seed and allocate each seed a unique label as an initial cluster. The remaining nodes are sorted in a decreasing order based on their densities.

#### 2.5.2 Label propagation

For each node outside clusters, we calculate the similarity ${S(n}_{i},C_{j})$ between the node$\;n_{i}\;$and each cluster $C_{j}$, i.e.,

(10) $$\begin{array}{c} S\left(n_{i},C_{j}\right)=\alpha \frac{\sum_{r\in \mathrm{ON}\left(i\right)\cap C_{j}}S_{\mathrm{ir}}+\sum_{r\in \mathrm{ON}\left(i\right)\cap U}\sum_{p\in \mathrm{ON}\left(r\right)\cap C_{j}}S_{\mathrm{rp}}}{\left|\mathrm{ON}\left(i\right)\cap C_{j}\right|+\sum_{r\in \mathrm{ON}\left(i\right)\cap U}\left|\mathrm{ON}\left(r\right)\cap C_{j}\right|}\\ +\left(1-\alpha \right)\frac{\sum_{r\in \mathrm{IN}\left(i\right)\cap C_{j}}S_{\mathrm{ri}}+\sum_{r\in \mathrm{IN}\left(i\right)\cap U}\sum_{p\in \mathrm{IN}\left(r\right)\cap C_{j}}S_{\mathrm{pr}}}{\left|\mathrm{IN}\left(i\right)\cap C_{j}\right|+\sum_{r\in \mathrm{IN}\left(i\right)\cap U}\left|\mathrm{IN}\left(r\right)\cap C_{j}\right|} \end{array}\;$$

where $\mathrm{ON}\left(i\right)$ and $\mathrm{IN}\left(i\right)$ represent the set of out-neighbors and in-neighbors of $n_{i}$ respectively, U the set of unlabeled nodes, and $S_{\mathrm{ij}}$ the similarity between $n_{i}$ and $n_{j}$. The first term on the right side of the equation can be viewed as the similarity from $n_{i}$ to $C_{j}$ obtained via dividing the sum of similarities along the paths from $n_{j}$ to each node in $C_{j}$ within a distance of two by the number of all paths. Similarly, the second term represents the similarity from $C_{j}$ to $n_{i}$ obtained via dividing the sum of similarities along paths from each node in $C_{j}$ to $n_{i}$ within a distance of two by the number of these paths. α is a combinatorial coefficient with a default value of 0.5.

For unlabeled nodes, we by $S\left(n_{i},U\right)$ denote the similarity between $n_{i}$ and U:

(11) $$\begin{array}{c} S\left(n_{i},U\right)=\alpha \frac{\sum_{r\in \mathrm{ON}\left(i\right)\cap U}S_{\mathrm{ir}}}{\left|\mathrm{ON}\left(i\right)\cap U\right|}+\left(1-\alpha \right)\frac{\sum_{r\in \mathrm{IN}\left(i\right)\cap U}S_{\mathrm{ri}}}{\left|\mathrm{IN}\left(i\right)\cap U\right|} \end{array}$$

We now let U exclude $n_{i}$, and $J={\mathrm{argmax}}_{j}S\left(n_{i},C_{j}\right)$. If ${S(n}_{i},C_{j})>S\left(n_{i},U\right)$, we add $n_{i}\;$to $C_{J}$ and label it; otherwise, we are of the opinion that there is insufficient information to assign a label to this node and the next node in U will be considered until all nodes have been processed in U.

#### 2.5.3 Pruning and merging

Pruning involves the removal of clusters that are deemed too small. After the removal, we prioritize merging the smaller clusters, ensuring that similar ones are combined into a single entity. (Algorithm 3, lines:17–19). By ${S(C}_{i},C_{j})$, we define the similarity between $C_{i}$ and $C_{j}$ as:

(12) $$\begin{array}{c} S\left(C_{i},C_{j}\right)=\sum_{r\in C_{i}}\left|\mathrm{ON}\left(r\right)\cap C_{j}\right|,\forall \left|C_{j}\right|>\left|C_{i}\right| \end{array}$$

If the maximum similarity ${S(C}_{i},C_{J})$ between $C_{i}$ and the larger cluster $C_{J}$ exceeds the number of edges within $C_{i}$, $C_{i}$ will be merged into $C_{J}$. Clusters that have assimilated other clusters cannot be amalgamated into a larger one.

#### 2.5.4 Cluster expansion

For each retained cluster $C_{j}$, we denote the average number of neighbors within the cluster as $A(C_{j})$. The similarity between each unlabeled node and a cluster $C_{j}$ is redefined as:

(13) $$\begin{array}{c} S\left(n_{i},C_{j}\right)=\left|\mathrm{ON}\left(i\right)\cap C_{j}\right|-A\left(C_{j}\right) \end{array}$$

The likelihood of a node belonging to the cluster increases with the size of the difference. If all the differences are less than 0, we can conclude that this node should not be included in any existing clusters. Instead, a new cluster should be created where this node will be placed. After labeling all nodes, the label propagation strategy is executed repeatedly until convergence.

Algorithm 3 Cluster Cells.

**Table:** 

### 2.6 Performance evaluation metrices

To measure the accuracy of the algorithm, we adopted four commonly used metrices: Adjusted Rand Index (ARI) [26], Normalized Mutual Information (NMI) [27], Fowlkes-Mallows Index (FMI) [28] and Jaccard Index (JI) [29]. Certainly, the arrangement of cluster labels will not change the score in any metric. The four indexes are calculated between the set I of identified cell clusters and the set A of annotated cell types. Let a be the number of correctly labeled cell pairs in the same cluster in both I and A; b be the number of cell pairs in the same group in A but in different clusters in I, c be the number of cell pairs in different groups in A but in the same cluster in I, and d be the number of cell pairs in different groups in both A and I. The four indexes are respectively defined as:

(14) $$\begin{array}{c} \mathrm{ARI}\left(I,A\right)=\frac{\mathrm{RI}-E\left(\mathrm{RI}\right)}{\max\left(\mathrm{RI}\right)-E\left(\mathrm{RI}\right)} \end{array}$$

(15) $$\begin{array}{c} \mathrm{NMI}\left(I,A\right)=\frac{2\mathrm{MI}}{H\left(I\right)+H\left(A\right)} \end{array}$$

(17) $$\begin{array}{c} \mathrm{FMI}\left(I,A\right)=\frac{a}{\sqrt{\left(a+b\right)\left(a+c\right)}} \end{array}$$

(18) $$\begin{array}{c} \mathrm{JI}\left(I,A\right)=\frac{a}{a+b+c} \end{array}$$

where RI is rand index of I and A defined as $\mathrm{RI}\left(I,A\right)=\frac{\left(a+d\right)}{c_{n}^{2}}$. Entropy H is the amount of uncertainty defined as $H\left(I\right)=-\sum_{i=1}^{\left|I\right|}P\left(i\right)\log\left(P\left(i\right)\right)$, where $P\left(i\right)=\frac{\left|I\right|}{N}$. The mutual information (MI) between I and A is calculated as $\mathrm{MI}\left(I,A\right)=\sum_{i=1}^{\left|I\right|}\sum_{j=1}^{\left|A\right|}P\left(i,j\right)\log\left(\frac{P\left(i,j\right)}{P\left(i\right)P^{\prime }\left(j\right)}\right)$.

### 2.7 Data availability

Twenty publicly available scRNA-seq datasets used for performance evaluation and analysis with annotated cell types are collected from https://hemberg-lab.github.io/scRNA.seq.datasets/, https://www.ncbi.nlm.nih.gov/geo/, https://www.ebi.ac.uk/ and R package “scRNAseq”. The number of cells ranges from 56 to 26,830. The details of twenty datasets are described in Table 1. The cell type labels are acquired from corresponding original publications, where labels are obtained via fluorescence-activated cell sorting (FACS), biological experiments (developmental timelines) and marker-based annotation. The datasets used for analysis from *Xenopus laevis* are accessible for download from https://bis.zju.edu.cn/XCL/.

**Table 1:** Details of twenty datasets.

Datasets	Accession IDs	Descriptions	# Cells	# Features	# Cell Types	Protocols	Ref.
Biase	GSE57249	Mouse Embryos	56	25,734	4	SMARTer	[30]
Yan	GSE36552	Human Embryos	90	20,214	6	Tang	[31]
Deng	GSE45719	Mouse Embryos	268	22,431	6	Smart-Seq2	[32]
Camp1	GSE75140	Human Brain	553	18,927	5	SMARTer	[33]
Lawlor	GSE86469	Human Pancreas	638	26,616	8	Fluidigm C1	[34]
Camp2	GSE81252	Human Liver	777	19,020	7	SMARTer	[35]
Xin	GSE81608	Human Pancreas	1492	39,851	4	SMARTer	[36]
Baronm	GSE84133	Mouse Pancreas	1886	14,878	13	inDrop	[37]
Muraro	GSE85241	Human Pancreas	2122	19,140	9	CEL-Seq2	[38]
Segerstolpe	E-MTAB-5061	Human Pancreas	2166	25,525	12	Smart-Seq2	[39]
Hermann	GSE109033	Mouse Spermatogenesis	2325	54,448	11	Drop-seq	[40]
Klein	GSE65525	Mouse ES	2717	24,175	4	inDrop	[41]
Romanov	GSE74672	Mouse Brain	2881	24,341	7	Drop-seq	[42]
Zeisel	GSE60361	Mouse Brain	3005	19,972	9	STRT-Seq UMI	[43]
Baronh	GSE84133	Human Pancreas	8569	20,125	14	inDrop	[37]
Aztekin	E-MTAB-7716	Xenopus laevis	13,199	31,535	46	10X Genomics	[44]
Chen	GSE87544	Mouse Brain	14,437	23,284	47	Drop-seq	[45]
Zilionis	GSE127465	Mouse Lung	15,939	28,205	7	inDrop	[46]
Campbell	GSE93374	Mouse Brain	20,921	26,774	20	Drop-seq	[47]
Shekhar	GSE81904	Mouse Retina	26,830	13,166	18	Drop-seq	[48]

## 3 Results

### 3.1 scQA effectively and efficiently clusters cells

**Fig. 2:**
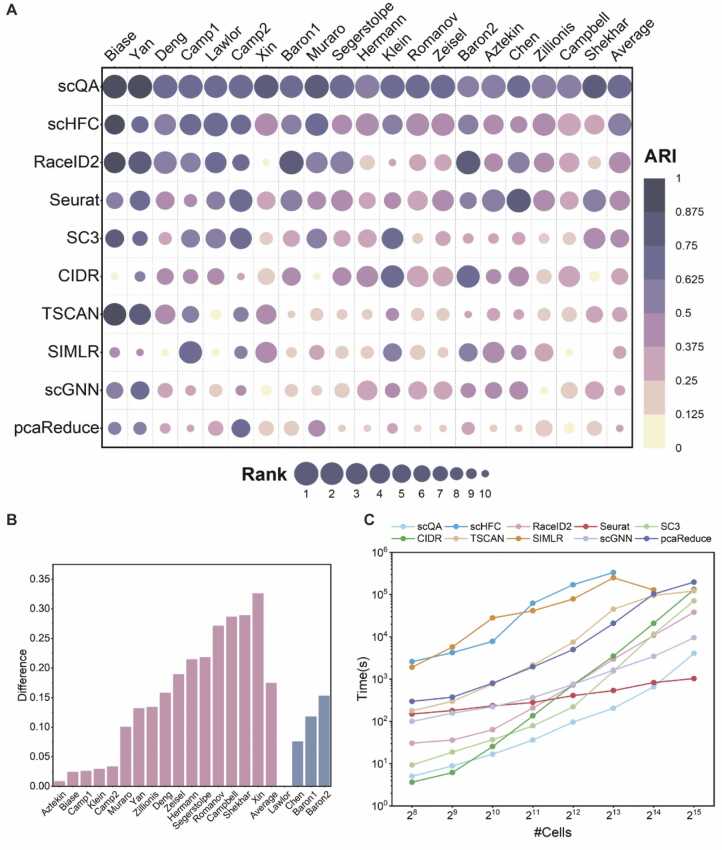
(**A**) ARI scores of scQA and compared algorithms on twenty datasets. The vacancy in the plot indicates that SIMLR failed to estimate the number of clusters and cluster cells on Shekhar dataset due to excessive memory usage. Average ARI for all datasets except the vacancy is displayed in the last column. (**B**) Difference of ARI between scQA and the second-best methods on 16 datasets where scQA achieved the highest ARI (pink bars) and difference of ARI between the best methods and scQA on 4 datasets where scQA failed to achieve the highest ARI (blue bars). (**C**) Comparison of distributions of running times against the number of cells. SIMLR failed to estimate the number of clusters and cluster cells on the dataset with 2^15^ cells due to excessive memory usage. Running times over 10^6^ s were not displayed.

To test scQA, we compared it with nine salient algorithms, including SC3, Seurat, CIDR, pcaReduce [49], SIMLR [50], TSCAN [51], RaceID2 [52], scGNN and scHFC [53] on twenty public datasets. The details of competitive algorithms are described in Supplementary Table S1 and Note S6. To systematically evaluate the accuracy of the competitive tools in recovering cell types, we utilized several evaluation metrics including ARI, FMI, NMI and JI with results shown in Fig. 2, Supplementary Fig. S1-S3 respectively. As shown in Fig. 2, scQA outperformed all compared algorithms on sixteen datasets, and was competitive with the best algorithm on the remaining four datasets (Fig. 2B). Especially, scQA was significantly superior to all the others on ten datasets with ARI score over 10% higher than the second-best algorithm and even more than 30% higher on Xin dataset. On average, scQA achieved ARI score of 0.71 (Supplementary Table S2), which surpassed second place (scHFC) by 17%. Further analysis regarding the clustering results can be found in the Supplementary Note S7.

We evaluated efficiency by comparing their distributions of running times against the numbers of cells (Fig. 2C). To test it, we generated data by varying the number of cells from 2^8^ to 2^15^ with number of genes fixed to 20,000 using a human liver immune scRNA-seq dataset [54]. All parameters of algorithms were set as before. As certain methods utilize multiple CPU cores for parallel computing whereas others rely on a single thread for clustering, we compared the amount of CPU time spent for each method. It's evident from the results that CIDR is the fastest method when the number of cells is below 1000, and scQA consistently exhibits significant efficiency, surpassing other methods for number of cells less than 2^15^. However, when the number of cells reaches 30,000, scQA becomes slightly slower than Seurat with both execution time orders of magnitude being 10^3^. Generally, scQA is faster compared to other high-performing methods, even when the number of cells exceeds 30,000, the runtime remains acceptable.

**Fig. 3:**
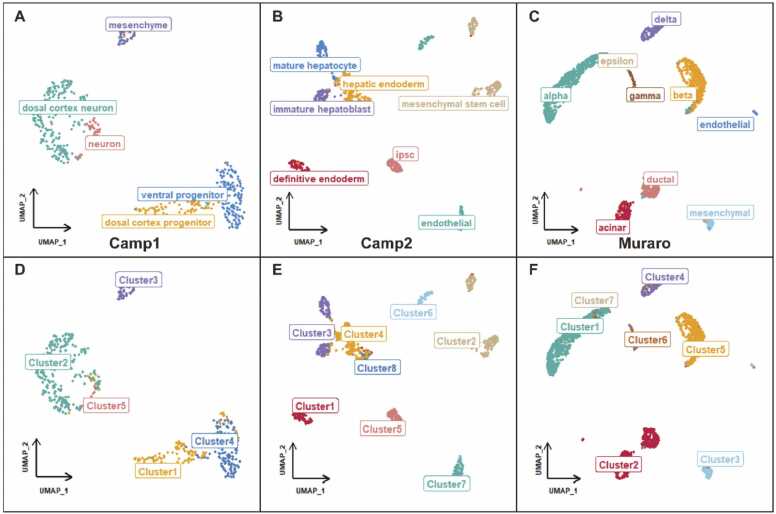
UMAP visualizations on three datasets labeled with original labels (**A**-**C**) and cluster labels (**D**-**F**) respectively.

The clustering results of scQA were illustrated through 2D visualization of embedded representations using uniform manifold approximation and projection (UMAP) (Fig. 3) and t-distributed stochastic neighbor embedding (t-SNE) (Supplementary Fig. S4). In Camp2 dataset, the cluster labeled with endothelial was further divided into two separate clusters (Cluster6 and Cluster7), because the UMAP plot indicated that the two subsets of endothelial cells were relatively distant from each other. As shown in Fig. 3C and F, endothelial cells and mesenchymal cells were widely separated in the original data but incorrectly grouped into a single cluster (Cluster3). One potential explanation for this is the number of cells belonging to endothelial cell type is extremely small to be ignored, leading to their inclusion in mesenchymal cells by merging step. The findings illustrate that scQA can accurately identify most cell types, with misclassified cells often appearing either too closely or distantly located in raw data visualizations.

### 3.2 Self-reliability of landmarks

In this section, we will demonstrate that genes exhibit characteristics of both internal similarity within a landmark and external dissimilarity among landmarks.

#### 3.2.1 Characteristics of landmarks hinting distinct cell types

**Fig. 4:**
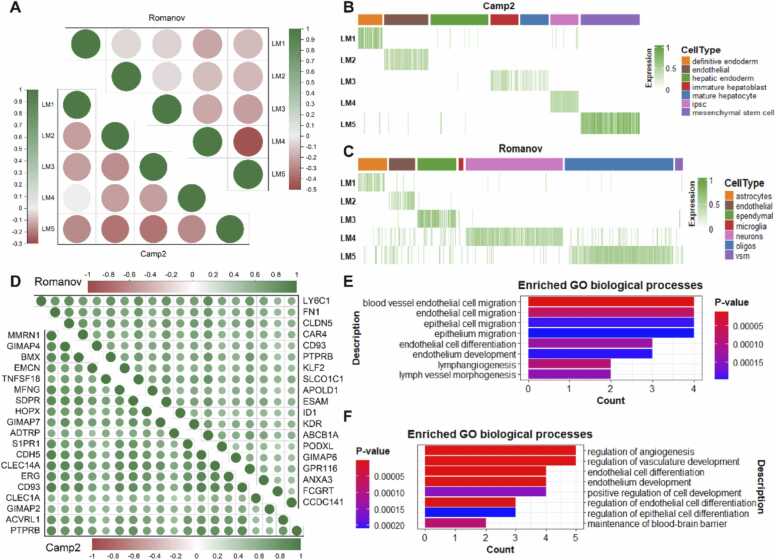
Similarity of genes in the same landmark and dissimilarity between landmarks. (**A**) Pearson correlation coefficient of five landmarks identified by scQA on Camp2 and Romanov datasets. (**B-C**) Heatmaps of five landmarks in Camp2 and Romanov datasets respectively. (**D**) Pearson correlation coefficient of genes in LM2 on Camp2 and Romanov datasets. (**E-F**) GO biological processes enriched in the gene set of LM2 in Camp2 and Romanov datasets respectively.

It is evident that distinguishing different cell types requires a variety of distinct and informative features. Therefore, we randomly selected five landmarks constructed in LC_2_ (column vectors in $Q_{2}$ matrix) each of them captured a specific cell type in Camp2 and Romanov datasets, and showed their non-relationship by calculating Pearson correlation coefficients. As shown in Fig. 4A, landmarks that capture different cell types are generally negatively correlated or irrelevant. This demonstrates that different landmarks can capture characteristic information for distinct cell types, and such information is mutually exclusive. Furthermore, Fig. 4B and C depict the expression patterns of the five landmarks, demonstrating the association of each landmark with specific cell types. It confirms that scQA can extract differentially expressed genes associated with these cell types, thereby enabling bidirectional clustering.

#### 3.2.2 Characteristics of genes within same landmark

To evaluate the ability of scQA in aggregating genes with high consistency, we calculated Pearson correlation coefficients among genes acquired from LM2 which corresponds to endothelia cell type in both Camp2 and Romanov datasets. Experiments investigating other landmarks are available in Supplementary Fig. S5 and S6.

As shown in Fig. 4D, all genes are positively correlated and the correlation coefficients are above 0.5 for almost all genes. Supplementary Fig. S7 illustrates the expression landscape of these genes, indicating that genes assigned to the same landmark exhibit remarkably similar expression patterns. Accordingly, scQA facilitates cell clustering by aggregating highly similar and informative genes based on expression values. We investigated biological resemblance by performing functional enrichment analysis using Gene Ontology (GO) biological processes on gene sets of the mentioned landmarks. Top eight terms were selected based on p-value. As shown in Fig. 4E, the enriched terms are directly related to the development of endothelial cells in Camp2 dataset. *ADTRP*, *CDH5*, *CLEC14A*, *ACVRL1* are enriched in the term “blood vessel endothelial cell migration” and served as markers for endothelia cell type [55], [56] except *ADTRP*. *ADTRP* regulates endothelial cell proliferation, apoptosis, migration and capillary tube formation, which shows endothelial-specific expression. Endothelial cells are also implicated in the formation of new blood vessels which known as angiogenesis, confirming genes enriched in terms “regulation of angiogenesis” and “regulation of vasculature development” in Romanov dataset (Fig. 4F). Taken together, quasi-trend-preserved genes selected by scQA have significant biological implications and interpretability.

### 3.3 External validation of landmarks

To further emphasize the significance of landmarks in cell clustering, we evaluate the reliability of landmarks through external information, concentrating on three aspects: differentially expressed genes (DEGs), hub genes and marker genes.

#### 3.3.1 Landmark genes reliably overlap with DEGs

**Fig. 5:**
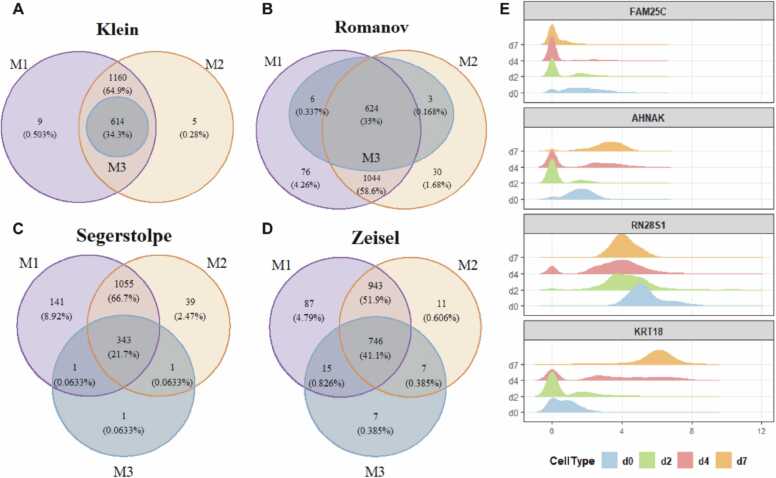
Comparison of DEGs and LMGs. (**A**-**D**) Venn diagrams of M1, M2 and M3 on four datasets respectively. (**E**) Expression values of feature genes extracted by scQA on Klein dataset.

We conducted a series of comparative experiments by comparing the DEGs and landmark genes (LMGs) identified by scQA on four datasets (Fig. 5A–D). To derive DEGs, we performed Wilcoxon Rank-Sum test implemented in the Seurat package along with Benjamini-Hochberg correction for adjusting p-value. Here, genes with the absolute value of logFC above 2 and adjusted p-value less than 0.01 were selected as DEGs. For convenience, we represent the set of DEGs with assigned reference labels and labels generated by scQA as M1 and M2 respectively, and use M3 to indicate the set of LMGs. For Klein dataset, the overlap odd (Supplementary Note S3) of M1 and M2 reached 99.2%, affirming the reliability of our clustering results compared to reference labels. And M3 was completely encompassed within the intersection of M1 and M2, demonstrating the robustness of our feature extraction strategy. Next, we performed Wilcoxon Rank-Sum tests on M3 in Klein and Zeisel datasets, the resulting top-ranked DEGs in each cell type were plotted (Fig. 5E, Supplementary Fig. S8). It is obvious in Fig. 5E, *Fam25c* and *Krt18* are up-regulated genes in the cell development of day 0 and day 7 respectively. Conversely, *Ahnak* and *Rn28s1* are down-regulated in the cell development of day 2 and day 4. Supplementary Fig. S8 illustrates the exclusive expression of certain genes in specific cell types, such as *Dlx6os1*, *Acsbg1*, *Myl9*, which serve as markers in [57], [58], [59], while *Hpca* exhibits diverse expression patterns across multiple cell types. The protein encoded *Hpca* is a member of neuron-specific calcium-binding proteins family found in the retina and brain, which plays an important role in various neurons and may express in multiple types of neurons. This phenomenon highlights genes characterized by binary expression patterns indeed recognize certain clusters, yet quantitative expression patterns of genes are crucial for making further distinction. It is essential to have both binary and quantitative expression patterns for deriving precise clusters.

#### 3.3.2 Landmark genes are generally hub genes

Weighted gene co-expression network analysis (WGCNA) can be used to detect highly correlated genes and genes with strong connections in the expression network are regarded as hub genes as well as considered functionally significant. The hub genes identified from different datasets are significantly overlapped with the landmark genes generated by scQA. As shown in Table 2, the number of hub genes were set to 100 and the number of LMGs respectively. In Camp1 dataset, 100 out of 2000 genes are regarded as hub genes, and 99 out of 100 hub genes can be found in 464 LMGs. To assess the significance, we conducted a hypergeometric test (Supplementary Note S4) with correction based on randomized trials. The results reveal that our method is highly significant for selecting landmark genes being hub genes, as the adjusted p-value is well below 0.01.

**Table 2:** Comparison of hub genes and LMGs in Camp1, Lawlor, Segerstolpe and Xin datasets.

Dataset	#hub genes	#landmark genes	#intersection	P-value	adjusted P
Camp1	100 464	464 464	99 348	1.827968 × 10^−65^ 1.961162 × 10^−180^	3.264543 × 10^−65^ 3.534021 × 10^−180^
Lawlor	100 460	460 460	69 241	8.749114 × 10^−24^ 1.338383 × 10^−58^	1.618149 × 10^−23^ 2.377996 × 10^−58^
Segerstolpe	100 346	346 346	98 240	2.140998 × 10^−77^ 2.66372 × 10^−138^	3.718499 × 10^−77^ 4.593871 × 10^−138^
Xin	100 174	174 174	57 82	6.761822 × 10^−38^ 6.438897 × 10^−48^	1.032473 × 10^−37^ 1.152201 × 10^−47^

#### 3.3.3 Landmark genes comprise marker genes

**Fig. 6:**
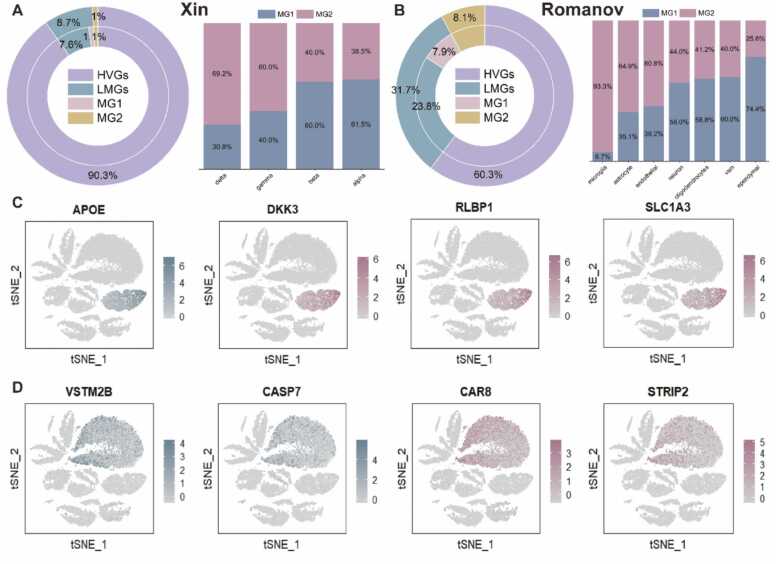
Comparison of marker genes and LMGs. (**A**, left, **B**, left) Doughnut chart of percentage of LMGs and marker genes (MG1, MG2) in HVGs in Xin and Romanov datasets respectively. (**A**, right, **B**, right) Percentage of marker genes within and outside of LMGs, denoted as MG1 and MG2 of all cell types in Xin and Romanov datasets respectively. (**C-D**) t-SNE visualizations of genes related to müller glia and rod bipolar cell types respectively in Shekhar dataset.

In the following, we evaluate the reliability of landmark genes by comparing them with marker genes. The marker genes employed were downloaded from Panglao DB (https://panglaodb.se/). Analyses were performed on 2000 HVGs. As depicted in Fig. 6A, the sum of MG1 and MG2 represents the number of marker genes, constituting 2.1% or precisely 42 genes in Xin dataset, in which 22 genes are covered in 174 LMGs. The analysis also reveals that scQA successfully identified marker genes for all cell types (Fig. 6A, right) and hypergeometric test shows that the results are remarkably significant in three out of four cell types (Supplementary Table S7). Fig. 6B of Romanov dataset demonstrates substantial variability in the proportion of marker genes found within LMGs across different cell types, varying from 74.4% to 6.7%. Notably, the cell type with the lowest percentage of marker genes in LMGs tends to be the cell type with the lowest number of cells in the dataset. For instance, in Romanov dataset, there are only 48 microglia cells, accounting for 1.6% of total 2881 cells.

Moreover, due to the limited record of marker genes in the database, we examined genes that belong to the same landmark groups as marker genes documented in the original literature of the Shekhar dataset. The Shekhar dataset includes five major cell types, one of which can be further subdivided into 14 subtypes (Supplementary Fig. S9, S10). We conducted our analysis on one major cell type and one subtype. *Apoe* is a major marker of müller glia (MG) cells in the retinal class used in the original literature. Therefore, we extracted several genes from the landmark group including *Apoe*. As illustrated in Fig. 6C, these genes exhibit similar expression patterns that are expressed almost exclusively in MG cells. *Apoe* exhibits a striking ability to express in all MG cells, while expresses in other cell types accounting for 51.8% of all expressed cells (Supplementary Table S7). The other three genes appear to exhibit more specificity with 74.8%, 74.2% and 83.9% of expressed MG cells in all expressed cells respectively. They are expressed in more than 96% MG cells and have been utilized as marker genes of MG cells in several literatures [60], [61], [62]. This finding suggests that scQA has the ability to identify similar marker genes, thereby combining them to create a single landmark. In the original study, *Vstm2b* and *Casp7* were identified as related to rod bipolar cells (RBCs) which is a subtype of bipolar cells with relatively large number of cells. We investigated the landmark group encompassing these two genes and identified multiple genes that exhibited similar expression patterns (Fig. 6D, Supplementary Fig. S11). Our analysis reveals that *Car8* manifests a higher percentage of being expressed (85.3%) in RBCs comparing with *Vstm2b* and *Casp7* (Supplementary Table S7), which has been confirmed as a marker gene in previous studies [63], [64]. Another gene, *Strip2*, also exhibits strong specificity in expression with a percentage of 90.7% of expressed cells being RBCs which is not documented in any reference as a marker gene. *Strip2* may be relevant to retinal development since a recent study highlighted the importance of *Strip1* in inner retina development [65] and both *Strip1* and *Strip2* play a role in regulating cell morphology and cytoskeletal organization and are paralogs to each other. The analysis indicates that scQA possesses the capability to create a landmark for similar marker genes identified, and facilitates the detection of new marker genes.

### 3.4 Analysis on single cells from *Xenopus laevis*

**Fig. 7:**
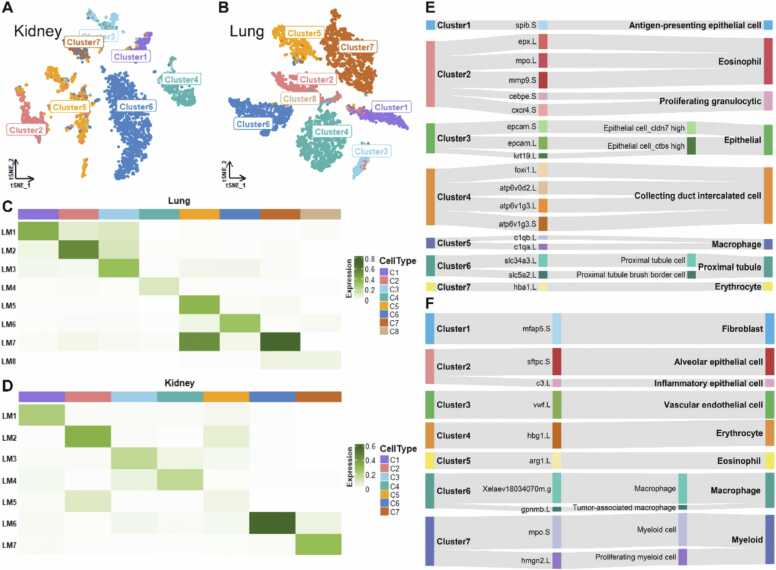
Clustering performance of kidney and lung datasets from *Xenopus laevis*. (**A**-**B**) t-SNE visualizations on two datasets. (**C-D**) Heatmaps of landmarks in lung and kidney datasets respectively. (**E**-**F**) Sankey plot showing marker genes for annotations for kidney and lung datasets respectively. The thickness of the line indicates the proportion of cells in the cluster that the gene is expressed in.

To affirm the versatility of scQA across various species, we conducted analyses on lung and kidney cells from *Xenopus laevis*. Clustering was performed separately for lung and kidney cells, as depicted in Fig. 7A, B. scQA divided kidney cells into 7 clusters and lung cells into 8 clusters. We assessed the clustering performance using three internal evaluation metrics, including Silhouette Coefficient, Calinski-Harabasz Index and Davies-Bouldin Index (Supplementary Table S9 and Supplementary Note S7). Both evaluation metrics and visualization collectively indicated that the clusters identified by scQA exhibited a relatively compact internal structure and dispersion externally. The most relevant landmark for each cell type is shown in Fig. 7C, D, wherein the expression pattern of each landmark closely correlates with a specific cluster.

Subsequently, we delved into analyzing the landmark genes. For the kidney dataset, we performed differential expression analysis on 2500 HVGs selected following reference [66], revealing 179 DEGs, following the same methodology as previously described. Among the 367 identified landmark genes in scQA, 176 were differentially expressed, encompassing 98.3% of the identified DEGs. Regarding the lung dataset, 229 DEGs were identified among the 2500 HVGs, and scQA pinpointed 217 DEGs within 418 landmark genes, covering approximately 94.8% of all DEGs. These findings are notably significant, and all identified DEGs can be referenced in Supplementary Data S1. Clusters were annotated using marker genes provided in reference [66] (Fig. 7E, F). In the kidney dataset, we accurately distinguished seven major cell types. Cluster6 contained two marker genes, *slc34a3. L* and *slc5a2. L*, designated as marker genes for proximal tubule cells and proximal tubule brush border cells, respectively. The proximal tubule brush border cell type can be considered as a subtype of proximal tubule cells. Similarly, in the lung dataset, seven major cell types were identified. However, Cluster8 lacked corresponding marker genes for annotation. Notably, although scQA identified eight clusters, Cluster8 contained only three cells, likely being outliers during clustering.

For fibroblasts in the lung dataset, in addition to the provided marker gene *mfap5. S*, we identified other genes with similar expression patterns (Supplementary Fig. S12). Five of these genes were highlighted: *col6a2. S*, *prg4. S*, *col1a1. L*, *fgl1. L*, and *c1s.L*. Among these, *COL6A2*, *PRG4*, *COL1A1* and *MFAP5* serve as markers for both human and mouse fibroblasts. *FGL1* is a protein coding gene and a member of the fibrinogen family. *c1s.L* is orthologous to human *C1S* (complement C1s), and its two orthologous genes, *C1s1* and *C1s2*, are used as marker genes for the fibroblasts in mouse. Regarding the eosinophil cell type within the kidney dataset, relevant marker genes included *mpo.L*, *epx.L*, and *mmp9. S*. *Xelaev18038242m.g* exhibited a specific expression pattern in both kidney and lung eosinophils, potentially acting as a marker gene associated with eosinophils.

### 3.5 Ablation study and parameter analysis

**Fig. 8:**
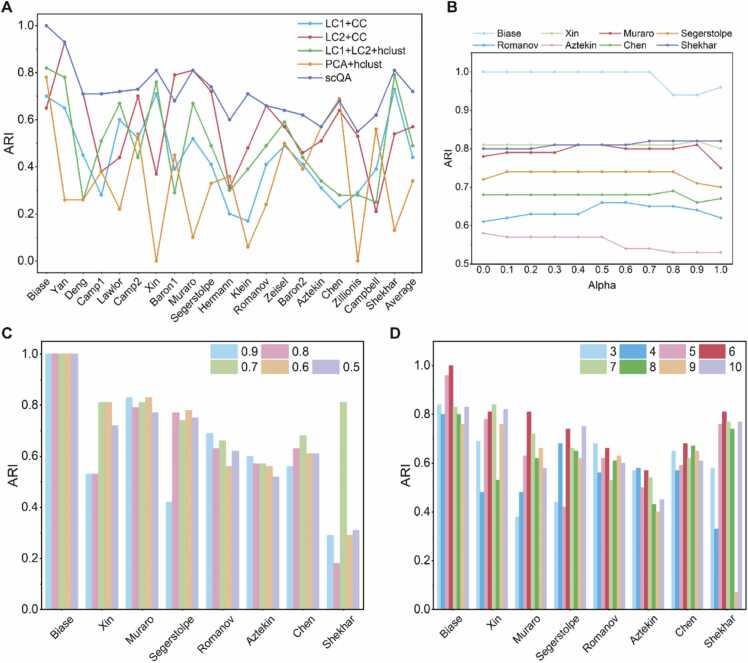
Ablation study and parameter analysis. (**A**) Comparison of distributions of ARI scores against twenty datasets of variants of scQA. (**B**) ARI scores of the parameter α ranging from 0 to 1 with a step size of 0.1 on eight datasets. (**C**) ARI scores of the parameter ρ ranging from 0.5 to 0.9 with a step size of 0.1 on eight datasets. (**D**) ARI scores of the parameter k ranging from 3 to 10 on eight datasets.

In order to investigate the crucial contribution of each module, we tested different variants of our proposed method to confirm the validity of each module. To illustrate that each of the two feature extraction modules is indispensable, we use each of them to extract features and perform clustering with CC separately. When using only LC_1_ for feature extraction, $Q_{1}$ was employed for seed generation and label propagation. When features were extracted utilizing LC_2_, binarized form of $Q_{2}$ was used for seed generation, while $Q_{2}$ was used for label propagation. As shown in Fig. 8, LC_1_+CC and LC_2_+CC were significantly inferior to scQA. In most cases, the clustering results achieved with features exclusively extracted by LC_2_ outperformed the results obtained from features extracted solely by LC_1_. This outcome is expected since LC_2_ extracts features with numerical information, while LC_1_ is limited to qualitative features. However, we have observed improved clustering results in several cases, such as Lawlor and Xin datasets, when only using the features extracted from LC_1_. This suggests that qualitative features are of vital importance and indeed have a beneficial impact on identifying cell types. It clearly demonstrates that the two feature extraction modules possess the capacity to extract distinct information, confirming that both qualitative and quantitative perspectives are essential. Integrating information from two perspectives can yield improved clustering results.

We evaluated the efficiency of CC by replacing it with hierarchical clustering. The number of clusters in hierarchical clustering was specified as the true number of cell types, providing a slight advantage to hierarchical clustering as we possess prior knowledge of the number of cell types. Despite this, the ARI scores of hierarchical clustering (LC_1_ + LC_2_ + hclust) were still much worse than scQA, which demonstrates the effectiveness of our ingenious label propagation strategy. In addition, we substituted our feature extraction module with PCA (the number of principal components was setting to 10), and notice that PCA achieved almost the worst clustering results. This serves as confirmation that our feature extraction method, which combines two perspectives, holds superior efficacy.

To conduct a thorough parameter analysis, this study offers a comprehensive examination on three essential parameters, which are the threshold parameter ρ in LC_1_ and LC_2_ modules, the number of bins k in LC_2_ module and a combinatorial coefficient α for calculating similarities between nodes and clusters in CC module.

To investigate the effect of ρ in feature extraction modules on clustering results, we tested on eight datasets with ρ ranging from 0.5 to 0.9 in increments of 0.1 (Fig. 8C). It is readily apparent that different datasets varied in their sensitivity to the threshold value. In general, the ARI scores indicate a trend of higher values within the medium range and lower values at both extremities. This situation is also reasonable because a higher threshold value increases the stringency of gene clustering, whereas a lower threshold can cause inaccurate clustering of genes. Another relatively important parameter in the feature extraction step is the number of bins. We conducted a comparative analysis for the same eight datasets with k ranging from 3 to 10 since k being equal to 2 is trivial which it is tantamount to the binarization of the data (Fig. 8D). It is noteworthy that the clustering results consistently performed better when k was set to 6, in which case the pre-grouping of genes was balanced, neither too subtle nor too coarse, making it more suitable for the subsequent clustering of genes.

During the process of cell clustering, the parameter α is utilized to appropriately balance the similarity from a single node to a cluster with the similarity from the cluster to the node for calculating the similarity in a directed graph. We carried out experiments on the above eight datasets with varying parametersα from 0 to 1 in the step of 0.1. When α=0, the similarity between a node and a cluster is determined solely by the directed edges from the cluster to the node, while α=1, the similarity is solely based on the directed edges from the node to the cluster. As shown in Fig. 8B, as α approaches 1 or 0, ARI scores drop significantly. It highlights the benefits of considering both directions of edges when calculating similarity for clustering. Furthermore, we analyzed the effect of different similarity metrics on the clustering results, the parameter for gene filtration during preprocessing and threshold of retained gene pairs for constructing gene similarity graph, the details of the experiments are shown in Supplementary Fig. S13, S14 and Note S5.

## 4 Discussion and conclusion

For the past decade, researchers have been working on analyzing single-cell RNA-seq datasets and one of the most significant challenges is to tackle dropouts appropriately. Increasing evidence has shown that dropouts should not be outright discarded at the outset of downstream analyses [21], [22], [23]. It has been observed that dropout events are primarily inherited from biological factors rather than technical ones in UMI-based data. Once cells are correctly grouped based on their heterogeneity, most dropouts will be eliminated. However, imputation or other preprocessing methods, which attempt to remove dropouts, may inevitably introduce biases. In this study, we introduce a novel algorithm that synergistically combines information from both qualitative and quantitative perspectives, which is the first time to integrate both perspectives for cell type identification as far as we know. By introducing the qualitative perspective, we extract the valuable information from dropout events, and we identify key genes by introducing the concept of quasi-trend-preserved genes, resulting in a significant enhancement in clustering accuracy. Specifically, we identify key genes as chunks of genes with analogous expression patterns, forming landmarks that have been validated to exhibit internal similarity and external dissimilarity, and confirmed to hold potential biological significance. Finally, qualitative and quantitative landmarks are utilized as features for creating cell clusters and identify genes associated with these cell types simultaneously using a novel label propagation strategy that incorporates seed generation, label propagation, pruning and merging and cluster expansion, enabling efficiently bidirectional clustering. Unlike conventional label propagation algorithm, our approach assigns labels exclusively to seeds rather than each cell [67]. The results unequivocally demonstrate scQA’s significant superiority over other popular methods and confirm the effectiveness and potential biological significance of the identified key genes through both external and internal validation. Furthermore, we demonstrate the capacity of scQA to detect new marker genes.

While scQA proves to be a dependable automated clustering tool for scRNA-seq datasets with various number of cells, it does have its limitations. In the pre-processing step, the retention of only 2000 HVGs for subsequent clustering analysis led to the exclusion of some informative genes (Supplementary Table S8). Therefore, incorporating priori information to retain informative genes could be explored as a potential option. Similar to other clustering methods that rely on similarity metrics, genes may be misclassified in LC_1_ and LC_2_ as the influence of the high dimensionality. Our future goal is to optimize the process for selecting HVGs, and incorporate scQA into the analysis of multi-omics data, capitalizing on its inherent structure that facilitates the fusion of two different types of information.

## Authors’ contributions

DL developed the method and wrote the software. DL and QLM designed the experiments; DL performed all analyses. GJL supervised the study. DL, QLM and GJL wrote the manuscript. All authors read and approved the final manuscript.

## Funding

This work was supported by 10.13039/501100001809National Science Foundation of China [11931008] and by Ministry of Science and Technology of China [2020YFA0712400].

## Declaration of Competing Interest

The authors declare that they have no known competing financial interests or personal relationships that could have appeared to influence the work reported in this paper.
