# Supplementary material for: scQA: A dual-perspective cell type identification model for single cell transcriptome data
Source: Comput Struct Biotechnol J. 2023 Dec 21;23:520–36. doi: 10.1016/j.csbj.2023.12.021 (PMC10791572; doi:10.1016/j.csbj.2023.12.021)
Supplement: Supplementary file 2 — Supplementary material. [file mmc2.docx]

**Supplementary Information of**

**scQA: A dual-perspective cell type identification model for single cell transcriptome data**

Di Li^1^, Qinglin Mei^2^, Guojun Li^1^*

^1^Research Center for Mathematics and Interdisciplinary Sciences, Shandong University, Qingdao 266237, China.

^2^MOE Key Laboratory of Bioinformatics, BNRIST Bioinformatics Division, Department of Automation, Tsinghua University, Beijing 100084, China.

*Correspondence: [gjli@sdu.edu.cn]

# Supplementary Tables

Table S1. Description of nine methods used for comparison.

Table S2. Comparison of ARI scores.

Table S3. Comparison of FMI scores.

Table S4. Comparison of NMI scores.

Table S5. Comparison of JI scores.

Table S6. Hypergeometric tests of marker genes within landmarks.

Table S7. Comparison of genes on the percentage of expressed cells.

Table S8. Comparison of the proportion of marker genes.

Table S9. Internal evaluation metrics for two *Xenopus* datasets.

# Supplementary Figures

Figure S1. Comparison of distributions of FMI scores.

Figure S2. Comparison of distributions of NMI scores.

Figure S3. Comparison of distributions of JI scores.

Figure S4. t-SNE visualizations on three datasets.

Figure S5. Dot plot of Pearson correlation coefficient of genes.

Figure S6. Dot plot of Pearson correlation coefficient of genes.

Figure S7. Heatmaps of landmark genes on two datasets.

Figure S8. Joy plot of feature genes.

Figure S9. t-SNE visualizations of major cell types in Shekhar dataset.

Figure S10. t-SNE visualizations of bipolar cells in Shekhar dataset.

Figure S11. t-SNE visualizations of genes related to rod bipolar cell type in Shekhar dataset.

Figure S12. t-SNE visualizations of genes related to *Xenopus laevis*.

Figure S13. Parameter analysis in the seed generation step.

Figure S14. Parameter analysis in pre-processing and constructing gene similarity graph.

Figure S15**.** Average precision and recall scores of the largest cell types.

Figure S16. t-SNE visualizations of cells labeled Blast in Deng dataset.

Figure S17. t-SNE visualizations of Beta cells in Lawlor dataset and Alpha cells in Xin dataset.

Figure S18. t-SNE visualizations of Erythrocyte 1 cells in Aztekin dataset

# Supplementary Notes

Note S1. Trend-preserved and quasi-trend-preserved.

Note S2. Complementary entropy.

Note S3. Calculation of overlap odds.

Note S4. Hypergeometric test.

Note S5. Similarity metrics.

Note S6. Comparison with other tools.

Note S7. Internal evaluation metrics

Note S8. Clustering analysis for large major cell types

# Supplementary Tables

**Table S1.** Description of nine methods used for comparison.

| Methods | Descriptions | Versions | | Sources | Ref. |
| --- | --- | --- | --- | --- | --- |
| SC3 | consensus method, PCA/ Laplacian matrix + k-means | | 1.20.0 | Bioconductor | [1] |
| Seurat | PCA + nearest neighbor graph | 4.0.3 | | CRAN | [2] |
| CIDR | zero-imputed similarities +PCA | 0.1.5 | | GitHub  (github.com/  VCCRI/CIDR) | [3] |
| Pca  Reduce | PCA + k-means | 1.0 | | GitHub  (github.com/  JustinaZ/pcaReduce) | [4] |
| SIMLR | multikernel construction | 1.18.0 | | Bioconductor | [5] |
| TSCAN | PCA + model-based | 1.30.0 | | Bioconductor | [6] |
| RaceID2 | k-medoids based on Pearson correlations | Mar 3, 2017 | | GitHub  (github.com/  dgrun/StemID) | [7] |
| scGNN | Deep learning  GCN+autoencoder | Nov 14,  2021 | | GitHub  (github.com/ juexinwang/scGNN) | [8] |
| scHFC | hybrid fuzzy FCM+GG | Jan 11, 2022 | | GitHub  (github.com/ WJ319/scHFC) | [9] |

**Table S2.** ARI scores against twenty datasets of the ten compared algorithms. The top three scores on each dataset are bolded.

|  | SC3 | CIDR | PCA | SIMLR | Seurat | TSCAN | RaceID2 | scHFC | scGNN | scQA |
| --- | --- | --- | --- | --- | --- | --- | --- | --- | --- | --- |
| Biase | 0.87 | 0.09 | 0.57 | 0.46 | 0.58 | **0.98** | **0.97** | 0.96 | 0.58 | **1.00** |
| Yan | 0.63 | 0.59 | 0.62 | 0.47 | 0.69 | **0.80** | **0.80** | 0.64 | 0.67 | **0.93** |
| Deng | 0.36 | 0.41 | 0.26 | 0.12 | 0.47 | 0.48 | **0.55** | **0.55** | 0.37 | **0.71** |
| Camp1 | 0.55 | 0.39 | 0.34 | **0.68** | 0.39 | 0.55 | 0.60 | **0.67** | 0.36 | **0.71** |
| Lawlor | 0.60 | 0.46 | 0.25 | 0.07 | 0.50 | 0.09 | **0.67** | **0.72** | 0.18 | **0.72** |
| Camp2 | **0.68** | 0.28 | 0.64 | 0.60 | **0.70** | 0.62 | 0.63 | 0.65 | 0.50 | **0.73** |
| Xin | 0.17 | 0.21 | 0.19 | **0.46** | 0.33 | 0.41 | 0.00 | **0.48** | 0.00 | **0.81** |
| Baron1 | 0.33 | 0.47 | 0.24 | 0.22 | **0.57** | 0.20 | **0.79** | 0.51 | 0.24 | **0.68** |
| Muraro | 0.52 | 0.12 | 0.41 | 0.33 | 0.46 | 0.23 | **0.58** | **0.71** | 0.22 | **0.81** |
| Segerstolpe | 0.26 | 0.39 | 0.13 | 0.18 | **0.49** | 0.14 | **0.53** | 0.47 | 0.18 | **0.74** |
| Hermann | 0.30 | **0.38** | 0.20 | 0.23 | 0.27 | 0.20 | 0.24 | **0.39** | 0.31 | **0.60** |
| Klein | **0.63** | **0.68** | 0.33 | 0.52 | 0.50 | 0.41 | 0.27 | 0.58 | 0.42 | **0.71** |
| Romanov | 0.23 | **0.32** | 0.15 | 0.25 | 0.30 | 0.24 | 0.27 | **0.38** | 0.28 | **0.66** |
| Zeisel | 0.32 | 0.37 | 0.16 | 0.20 | **0.41** | 0.20 | 0.32 | **0.45** | 0.33 | **0.64** |
| Baron2 | 0.27 | **0.73** | 0.36 | 0.54 | 0.53 | 0.26 | **0.77** | 0.59 | 0.46 | **0.62** |
| Aztekin | 0.29 | 0.42 | 0.21 | **0.49** | **0.56** | 0.31 | **0.49** | **0.49** | 0.43 | **0.57** |
| Chen | 0.35 | 0.50 | 0.25 | 0.42 | **0.75** | 0.32 | **0.61** | 0.45 | 0.46 | **0.68** |
| Zillionis | 0.15 | 0.17 | 0.21 | 0.35 | **0.41** | 0.17 | 0.40 | **0.42** | 0.05 | **0.55** |
| Campbell | 0.13 | **0.28** | 0.12 | 0.10 | 0.26 | 0.21 | 0.26 | **0.33** | 0.23 | **0.62** |
| Shekhar | 0.40 | 0.07 | 0.25 |  | **0.52** | 0.28 | 0.21 | **0.37** | 0.33 | **0.81** |
| Average | 0.40 | 0.37 | 0.29 | 0.35 | 0.48 | 0.35 | **0.50** | **0.54** | 0.33 | **0.71** |

**Table S3.** FMI scores against twenty datasets of the ten compared algorithms. The top three scores on each dataset are bolded.

|  | SC3 | CIDR | PCA | SIMLR | Seurat | TSCAN | RaceID2 | scHFC | scGNN | scQA |
| --- | --- | --- | --- | --- | --- | --- | --- | --- | --- | --- |
| Biase | 0.91 | 0.46 | 0.69 | 0.61 | 0.76 | **0.98** | **0.98** | 0.97 | 0.76 | **1.00** |
| Yan | 0.74 | 0.69 | 0.69 | 0.58 | 0.79 | **0.84** | **0.85** | 0.71 | 0.76 | **0.95** |
| Deng | 0.52 | 0.57 | 0.44 | 0.30 | 0.62 | 0.62 | **0.68** | **0.68** | 0.54 | **0.83** |
| Camp1 | 0.67 | 0.56 | 0.49 | **0.78** | 0.53 | 0.66 | 0.72 | **0.75** | 0.51 | **0.79** |
| Lawlor | 0.72 | 0.61 | 0.43 | 0.22 | 0.64 | 0.31 | **0.77** | **0.80** | 0.40 | **0.82** |
| Camp2 | **0.74** | 0.41 | 0.71 | 0.67 | **0.75** | 0.68 | 0.69 | 0.72 | 0.58 | **0.77** |
| Xin | 0.40 | 0.48 | 0.42 | 0.66 | 0.56 | 0.62 | **0.68** | **0.68** | 0.29 | **0.91** |
| Baron1 | 0.50 | 0.60 | 0.40 | 0.39 | **0.69** | 0.36 | **0.86** | 0.63 | 0.40 | **0.79** |
| Muraro | 0.63 | 0.29 | 0.54 | 0.48 | 0.58 | 0.39 | **0.71** | **0.77** | 0.39 | **0.85** |
| Segerstolpe | 0.41 | 0.54 | 0.30 | 0.33 | **0.61** | 0.27 | **0.64** | 0.58 | 0.34 | **0.81** |
| Hermann | 0.48 | **0.60** | 0.38 | 0.41 | 0.45 | 0.39 | 0.48 | **0.55** | 0.48 | **0.73** |
| Klein | **0.73** | **0.77** | 0.49 | 0.64 | 0.63 | 0.56 | 0.43 | 0.69 | 0.56 | **0.79** |
| Romanov | 0.38 | **0.48** | 0.30 | 0.40 | 0.46 | 0.39 | 0.42 | **0.52** | 0.42 | **0.74** |
| Zeisel | 0.46 | **0.57** | 0.29 | 0.35 | 0.54 | 0.35 | 0.45 | **0.55** | 0.45 | **0.74** |
| Baron2 | 0.42 | **0.79** | 0.47 | 0.63 | 0.64 | 0.38 | **0.82** | 0.66 | 0.56 | **0.72** |
| Aztekin | 0.38 | 0.57 | 0.29 | 0.55 | **0.61** | 0.39 | **0.61** | 0.56 | 0.48 | **0.64** |
| Chen | 0.45 | 0.61 | 0.36 | 0.49 | **0.78** | 0.40 | **0.68** | 0.52 | 0.52 | **0.72** |
| Zillionis | 0.33 | 0.40 | 0.39 | 0.53 | **0.58** | 0.35 | 0.56 | **0.58** | 0.25 | **0.69** |
| Campbell | 0.29 | **0.57** | 0.30 | 0.25 | 0.42 | 0.38 | 0.47 | **0.50** | 0.40 | **0.77** |
| Shekhar | **0.52** | 0.34 | 0.37 |  | **0.63** | 0.42 | 0.36 | 0.48 | 0.45 | **0.85** |
| Average | 0.53 | 0.54 | 0.44 | 0.49 | 0.61 | 0.49 | **0.64** | **0.65** | 0.48 | **0.80** |

**Table S4.** NMI scores against twenty datasets of the ten compared algorithms. The top three scores on each dataset are bolded.

|  | SC3 | CIDR | PCA | SIMLR | Seurat | TSCAN | RaceID2 | scHFC | scGNN | scQA |
| --- | --- | --- | --- | --- | --- | --- | --- | --- | --- | --- |
| Biase | 0.86 | 0.33 | 0.70 | 0.73 | 0.70 | **0.96** | **0.95** | 0.93 | 0.70 | **1.00** |
| Yan | 0.72 | 0.73 | 0.76 | 0.73 | 0.80 | **0.84** | **0.86** | 0.79 | 0.75 | **0.93** |
| Deng | 0.68 | 0.72 | 0.52 | 0.56 | **0.73** | 0.68 | **0.75** | **0.74** | 0.54 | 0.70 |
| Camp1 | **0.67** | 0.46 | 0.58 | **0.68** | 0.60 | 0.58 | 0.58 | **0.69** | 0.43 | 0.66 |
| Lawlor | **0.67** | 0.56 | 0.52 | 0.43 | 0.64 | 0.21 | **0.68** | **0.67** | 0.32 | 0.62 |
| Camp2 | **0.83** | 0.50 | **0.81** | 0.80 | **0.84** | 0.80 | 0.80 | 0.79 | 0.68 | **0.81** |
| Xin | 0.36 | 0.23 | 0.48 | **0.59** | **0.59** | 0.56 | 0.00 | **0.67** | 0.01 | **0.72** |
| Baron1 | **0.68** | 0.51 | 0.53 | 0.62 | **0.80** | 0.49 | **0.74** | 0.66 | 0.54 | 0.63 |
| Muraro | **0.75** | 0.35 | 0.65 | 0.66 | 0.73 | 0.40 | 0.57 | **0.77** | 0.39 | **0.76** |
| Segerstolpe | 0.61 | 0.51 | 0.43 | 0.53 | **0.77** | 0.34 | **0.75** | 0.59 | 0.36 | **0.73** |
| Hermann | **0.58** | **0.57** | 0.42 | 0.50 | **0.57** | 0.49 | 0.46 | 0.56 | 0.54 | **0.64** |
| Klein | **0.76** | 0.66 | 0.56 | 0.68 | **0.72** | 0.64 | 0.36 | **0.72** | 0.59 | 0.69 |
| Romanov | 0.43 | 0.34 | 0.24 | 0.47 | **0.60** | 0.43 | 0.38 | **0.49** | 0.41 | **0.58** |
| Zeisel | 0.58 | 0.47 | 0.30 | 0.53 | **0.65** | 0.30 | 0.46 | **0.59** | 0.51 | **0.67** |
| Baron2 | 0.61 | 0.72 | 0.57 | **0.75** | **0.77** | 0.50 | **0.77** | 0.71 | 0.59 | 0.63 |
| Aztekin | 0.63 | 0.58 | 0.40 | **0.66** | **0.74** | 0.61 | 0.59 | 0.63 | 0.60 | **0.67** |
| Chen | 0.60 | 0.56 | 0.43 | 0.62 | **0.72** | 0.56 | **0.62** | 0.59 | 0.59 | **0.64** |
| Zillionis | 0.46 | 0.41 | 0.41 | 0.54 | **0.67** | 0.48 | 0.52 | **0.55** | 0.07 | **0.55** |
| Campbell | **0.53** | 0.39 | 0.30 | 0.47 | **0.64** | 0.49 | 0.40 | 0.49 | 0.50 | **0.56** |
| Shekhar | **0.60** | 0.25 | 0.38 |  | **0.82** | **0.60** | 0.30 | 0.54 | 0.45 | 0.51 |
| Average | 0.63 | 0.49 | 0.50 | 0.61 | **0.70** | 0.55 | 0.58 | **0.66** | 0.48 | **0.69** |

**Table S5.** JI scores against twenty datasets of the ten compared algorithms. The top three scores on each dataset are bolded.

|  | SC3 | CIDR | PCA | SIMLR | Seurat | TSCAN | RaceID2 | scHFC | scGNN | scQA |
| --- | --- | --- | --- | --- | --- | --- | --- | --- | --- | --- |
| Biase | 0.83 | 0.28 | 0.52 | 0.38 | 0.58 | **0.97** | **0.95** | **0.95** | 0.58 | **1.00** |
| Yan | 0.57 | 0.52 | 0.53 | 0.37 | 0.63 | **0.73** | **0.73** | 0.55 | 0.60 | **0.90** |
| Deng | 0.32 | 0.38 | 0.24 | 0.09 | 0.41 | 0.42 | **0.50** | **0.51** | 0.36 | **0.69** |
| Camp1 | 0.47 | 0.38 | 0.28 | **0.63** | 0.32 | 0.48 | 0.56 | **0.60** | 0.33 | **0.66** |
| Lawlor | 0.54 | 0.42 | 0.22 | 0.08 | 0.44 | 0.17 | **0.61** | **0.66** | 0.25 | **0.69** |
| Camp2 | **0.56** | 0.26 | 0.52 | 0.47 | **0.58** | 0.50 | 0.51 | **0.56** | 0.40 | **0.63** |
| Xin | 0.20 | 0.30 | 0.19 | 0.45 | 0.32 | 0.40 | **0.46** | **0.47** | 0.15 | **0.82** |
| Baron1 | 0.26 | 0.42 | 0.22 | 0.17 | **0.49** | 0.20 | **0.75** | 0.44 | 0.20 | **0.64** |
| Muraro | 0.41 | 0.17 | 0.33 | 0.25 | 0.36 | 0.24 | **0.53** | **0.62** | 0.24 | **0.74** |
| Segerstolpe | 0.19 | 0.37 | 0.18 | 0.13 | 0.39 | 0.13 | **0.43** | **0.40** | 0.20 | **0.68** |
| Hermann | 0.26 | **0.42** | 0.19 | 0.20 | 0.22 | 0.17 | 0.32 | **0.35** | 0.26 | **0.58** |
| Klein | **0.54** | **0.61** | 0.27 | 0.44 | 0.41 | 0.33 | 0.26 | 0.51 | 0.36 | **0.65** |
| Romanov | 0.18 | **0.31** | 0.16 | 0.20 | 0.23 | 0.19 | 0.25 | **0.34** | 0.25 | **0.59** |
| Zeisel | 0.24 | **0.37** | 0.15 | 0.15 | 0.31 | 0.21 | 0.29 | **0.38** | 0.28 | **0.57** |
| Baron2 | 0.19 | **0.65** | 0.29 | 0.44 | 0.42 | 0.20 | **0.69** | 0.49 | 0.38 | **0.55** |
| Aztekin | 0.19 | 0.33 | 0.17 | 0.37 | **0.43** | 0.21 | **0.39** | 0.38 | 0.32 | **0.45** |
| Chen | 0.24 | 0.40 | 0.21 | 0.31 | **0.64** | 0.22 | **0.50** | 0.35 | 0.35 | **0.56** |
| Zillionis | 0.12 | 0.24 | 0.19 | 0.29 | 0.34 | 0.14 | **0.38** | **0.38** | 0.13 | **0.52** |
| Campbell | 0.11 | **0.37** | 0.17 | 0.08 | 0.22 | 0.19 | 0.31 | **0.32** | 0.23 | **0.60** |
| Shekhar | 0.30 | 0.19 | 0.21 |  | **0.41** | 0.20 | 0.22 | **0.31** | 0.28 | **0.74** |
| Average | 0.34 | 0.37 | 0.26 | 0.29 | 0.41 | 0.32 | **0.48** | **0.48** | 0.31 | **0.66** |

**Table S6.** Marker genes in landmark genes identified by scQA of Xin and Romanov datasets and p-values by conducting hypergeometric tests. Significant p-values are bolded. Two cell types may share the same marker gene, the total number of marker genes may be less than the sum of the number of marker genes in each cell type.

| **Dataset: Xin** | | | | |
| --- | --- | --- | --- | --- |
| Cell types | #markers | #markers in LM | P-value | Adjusted P-value |
| beta | 15 | 9 | **2.477342×10^-6^** | **3.565238×10^-6^** |
| alpha | 13 | 8 | **7.409128×10^-7^** | **9.932151×10^-7^** |
| gamma | 13 | 4 | **7.65467×10^-3^** | **7.65467×10^-3^** |
| delta | 10 | 4 | 2.118051×10^-2^ | 3.04817×10^-2^ |
| all | 42 | 22 | **1.456747×10^-13^** | **2.015887×10^-13^** |
| **Dataset: Romanov** | | | | |
| Cell types | #markers | #markers in LM | P-value | Adjusted P-value |
| ependymal | 39 | 29 | **3.929741×10^-8^** | **6.465419×10^-8^** |
| vsm | 5 | 3 | 1.853254×10^-1^ | 2.177297×10^-1^ |
| oligodendrocyte | 51 | 30 | **4.564817×10^-5^** | **7.999142×10^-5^** |
| neuron | 84 | 47 | **2.190227×10^-6^** | **3.664409×10^-6^** |
| endothelial | 97 | 38 | 6.578843×10^-2^ | 1.093981×10^-1^ |
| astrocyte | 37 | 13 | 3.818513×10^-1^ | 5.782224×10^-1^ |
| microglia | 15 | 1 | 9.967607×10^-1^ | 1 |
| all | 318 | 157 | **4.534279×10^-13^** | **8.128871×10^-13^** |

**Table S7.** The proportion of expressed cells of genes that are in the same landmark as marker gene recorded in the original literature associated with müller glia (MG) cells and rod bipolar cells (RBCs) in Shekhar dataset.

| **Cell type: müller glia cells (2945)** | | | | |
| --- | --- | --- | --- | --- |
|  | #expressed cells | #expressed MG cells | Percentage of expressed MG in all MG cells | Percentage of expressed MG in all expressed cells |
| APOE | 6109 | 2945 | 1 | 0.482 |
| Dkk3 | 3900 | 2919 | 0.991 | 0.748 |
| RLBP1 | 3911 | 2902 | 0.985 | 0.742 |
| SLC1A3 | 3377 | 2833 | 0.962 | 0.839 |
| **Cell type: rod bipolar cells (10888)** | | | | |
|  | #expressed cells | #expressed RBC | Percentage of expressed RBC in all RBC | Percentage of expressed RBC in all expressed cells |
| VSTM2B | 7901 | 7071 | 0.649 | 0.895 |
| CAR8 | 10909 | 9286 | 0.853 | 0.851 |
| ADRB1 | 5118 | 4722 | 0.434 | 0.923 |
| STRIP2 | 6699 | 6077 | 0.558 | 0.907 |
| KCNE2 | 4832 | 4515 | 0.415 | 0.934 |
| SEBOX | 7197 | 6316 | 0.580 | 0.878 |
| CASP7 | 4710 | 4347 | 0.399 | 0.923 |
| ADAMTS5 | 5880 | 4733 | 0.435 | 0.805 |

**Table S8.** The proportion of marker genes included in the 2,000 genes selected in the pre-processing step to marker genes included in the original dataset. Since two cell types may share the same marker gene, the total number of marker genes may be less than the sum of the number of marker genes in each cell type.

| **Dataset: Xin** | | | |
| --- | --- | --- | --- |
| Cell Types | #markers | #markers in HVGs | proportion |
| beta | 52 | 15 | 28.8% |
| alpha | 46 | 13 | 28.3% |
| gamma | 34 | 13 | 38.2% |
| delta | 28 | 10 | 35.7% |
| all | 91 | 42 | 46.2% |
| **Dataset: Baron1** | | | |
| Cell Types | #markers | #markers in HVGs | proportion |
| beta | 54 | 16 | 29.6% |
| ductal | 43 | 22 | 51.2% |
| delta | 34 | 12 | 35.3% |
| gamma | 29 | 7 | 24.1% |
| alpha | 48 | 21 | 43.8% |
| schwann | 16 | 1 | 6.3% |
| T cell | 107 | 17 | 15.9% |
| B cell | 110 | 18 | 16.4% |
| macrophages | 153 | 31 | 20.3% |
| stellate | 29 | 13 | 44.8% |
| endothelial | 195 | 99 | 50.8% |
| all | 730 | 219 | 30% |

**Table S9.** Internal evaluation metrics for two *Xenopus* datasets.

| Data | Silhouette Coefficient | | Calinski-Harabasz Index | Davies-Bouldin Index |
| --- | --- | --- | --- | --- |
|  | Mean | Median |  |  |
| Kidney | 0.32896 | 0.49010 | 511.5133 | 0.4136393 |
| Lung | 0.4194 | 0.4470 | 1120.371 | 0.5023391 |

# Supplementary Figures

**
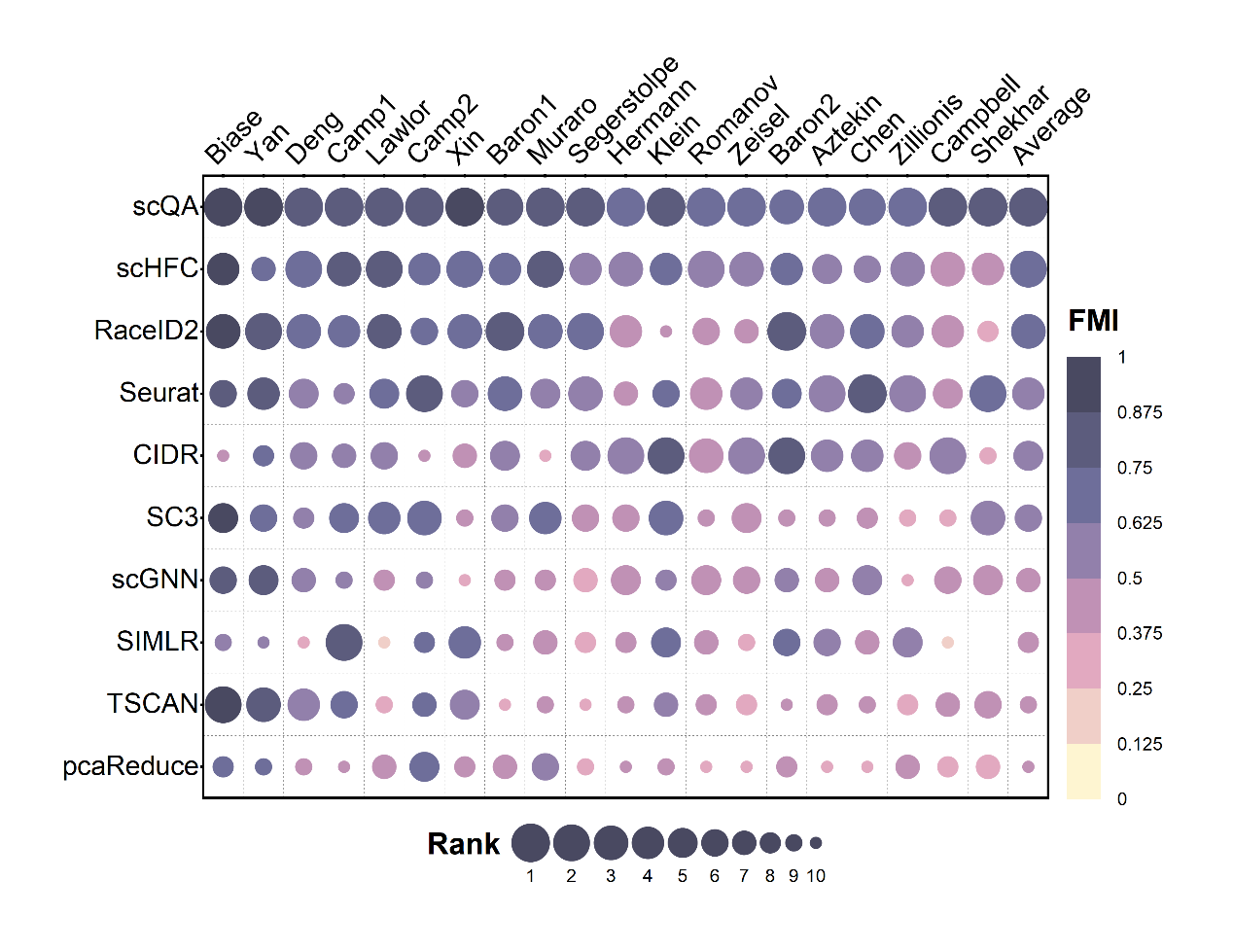
**

**Figure S1.** Comparison of distributions of FMI scores against twenty datasets of the ten compared algorithms. Names of the datasets are represented on the x-axis, and methods are shown on the y-axis. Each scatter point reflects the clustering performance with color representing the FMI score and size indicating the rank. The vacancy in the plot indicates that SIMLR failed to estimate the number of clusters and cluster cells on Shekhar dataset due to excessive memory usage. Average FMI for all datasets except the vacancy is displayed in the last column.


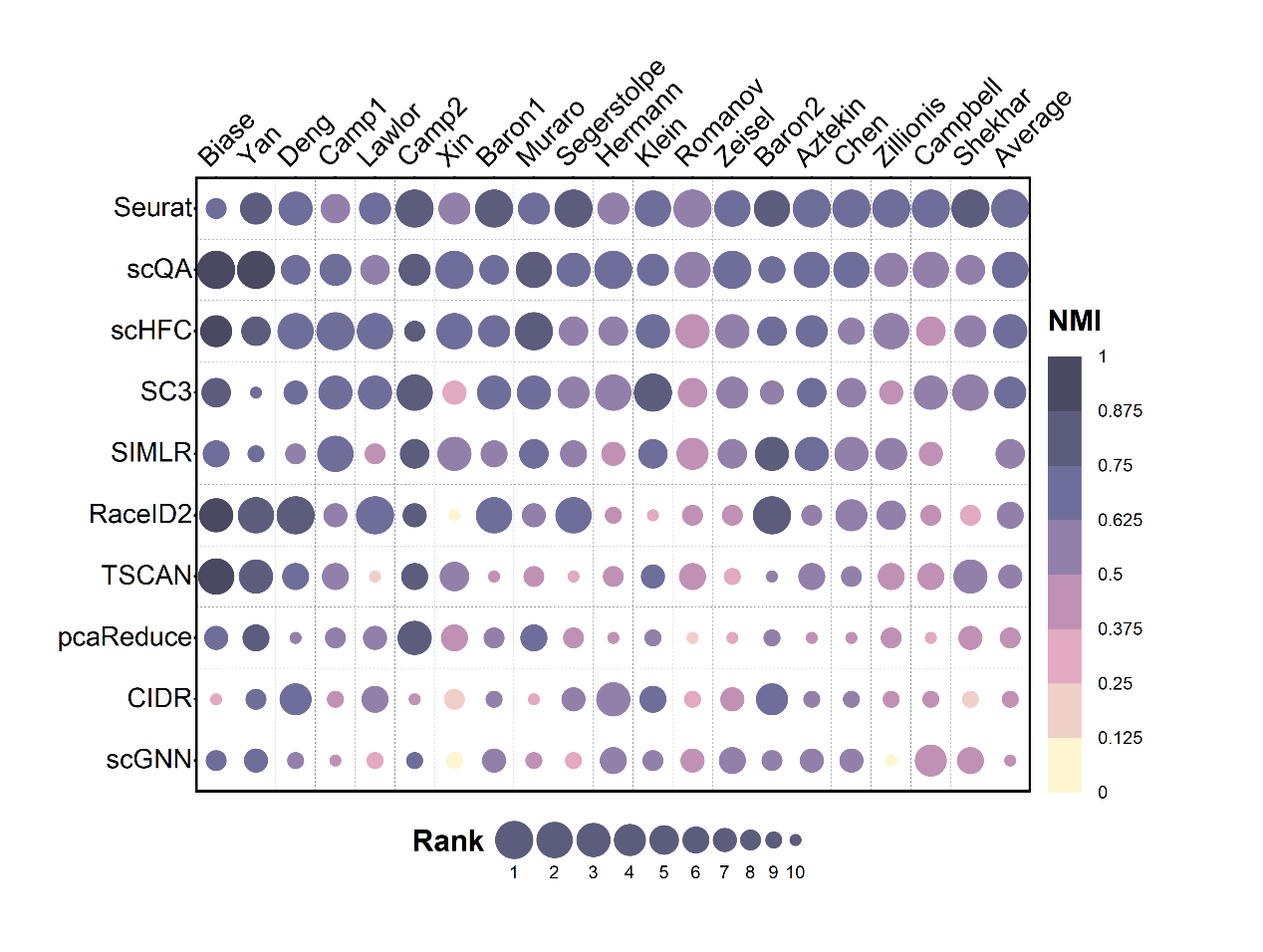


**Figure S2.** Comparison of distributions of NMI scores against twenty datasets of the ten compared algorithms. Names of the datasets are represented on the x-axis, and methods are shown on the y-axis. Each scatter point reflects the clustering performance with color representing the NMI score and size indicating the rank. The vacancy in the plot indicates that SIMLR failed to estimate the number of clusters and cluster cells on Shekhar dataset due to excessive memory usage. Average NMI for all datasets except the vacancy is displayed in the last column.


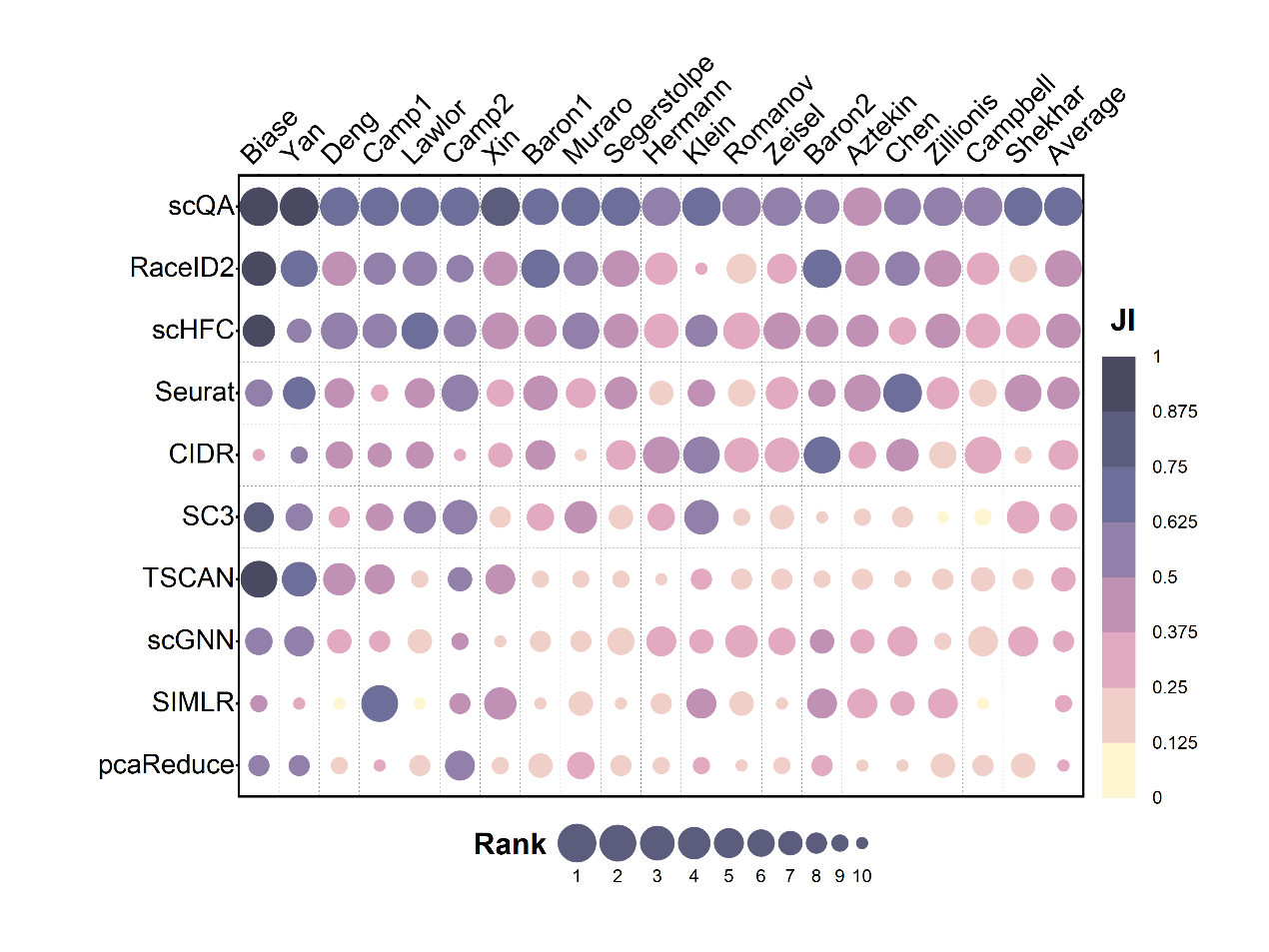


**Figure S3.** Comparison of distributions of JI scores against twenty datasets of the ten compared algorithms. Names of the datasets are represented on the x-axis, and methods are shown on the y-axis. Each scatter point reflects the clustering performance with color representing the JI score and size indicating the rank. The vacancy in the plot indicates that SIMLR failed to estimate the number of clusters and cluster cells on Shekhar dataset due to excessive memory usage. Average JI for all datasets except the vacancy is displayed in the last column.


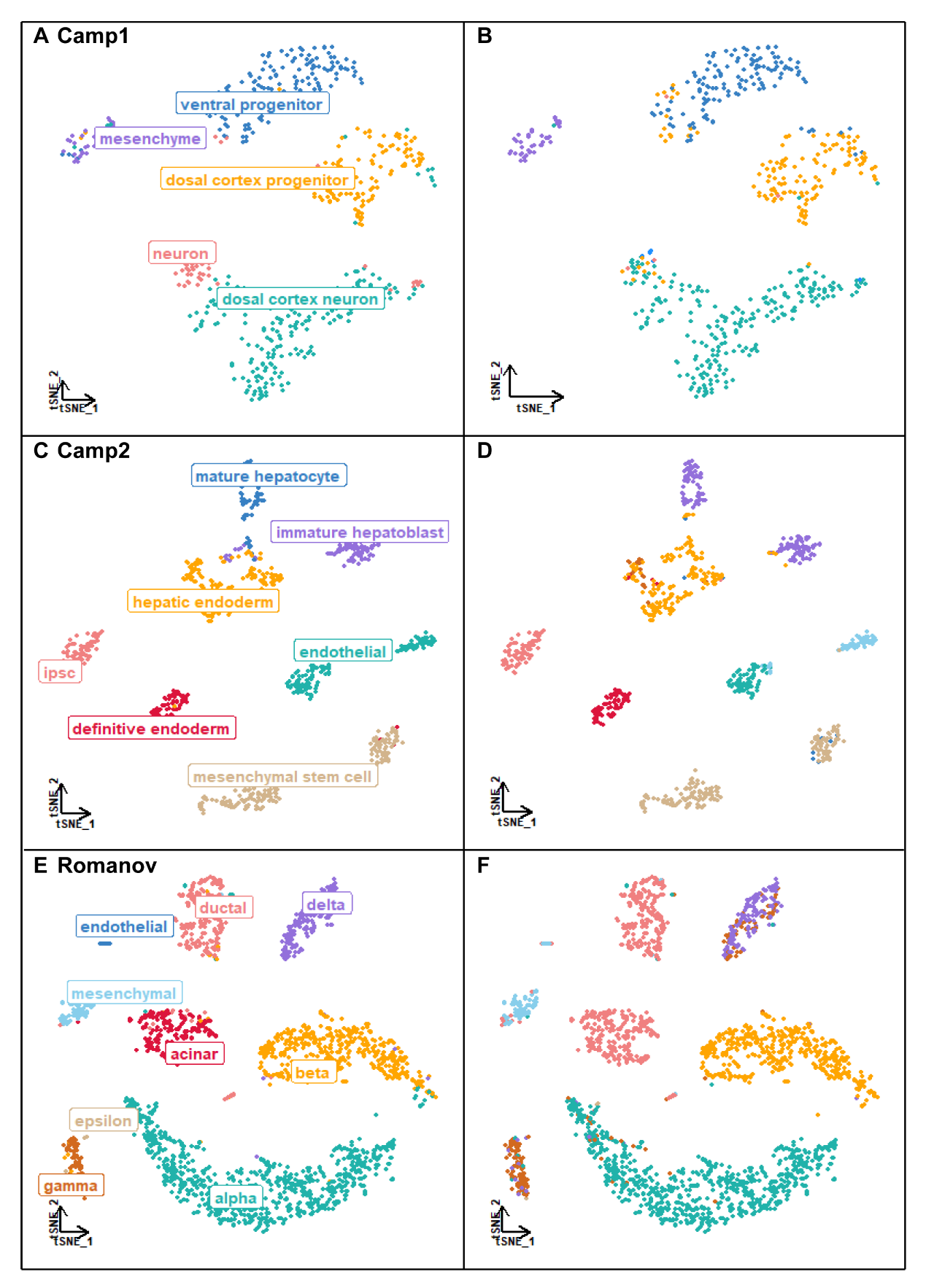


**Figure S4.** t-SNE visualizations on Camp1 dataset (top), Camp2 dataset (middle) and Muraro dataset (bottom) labeled with original labels (left) and cluster labels (right) respectively.


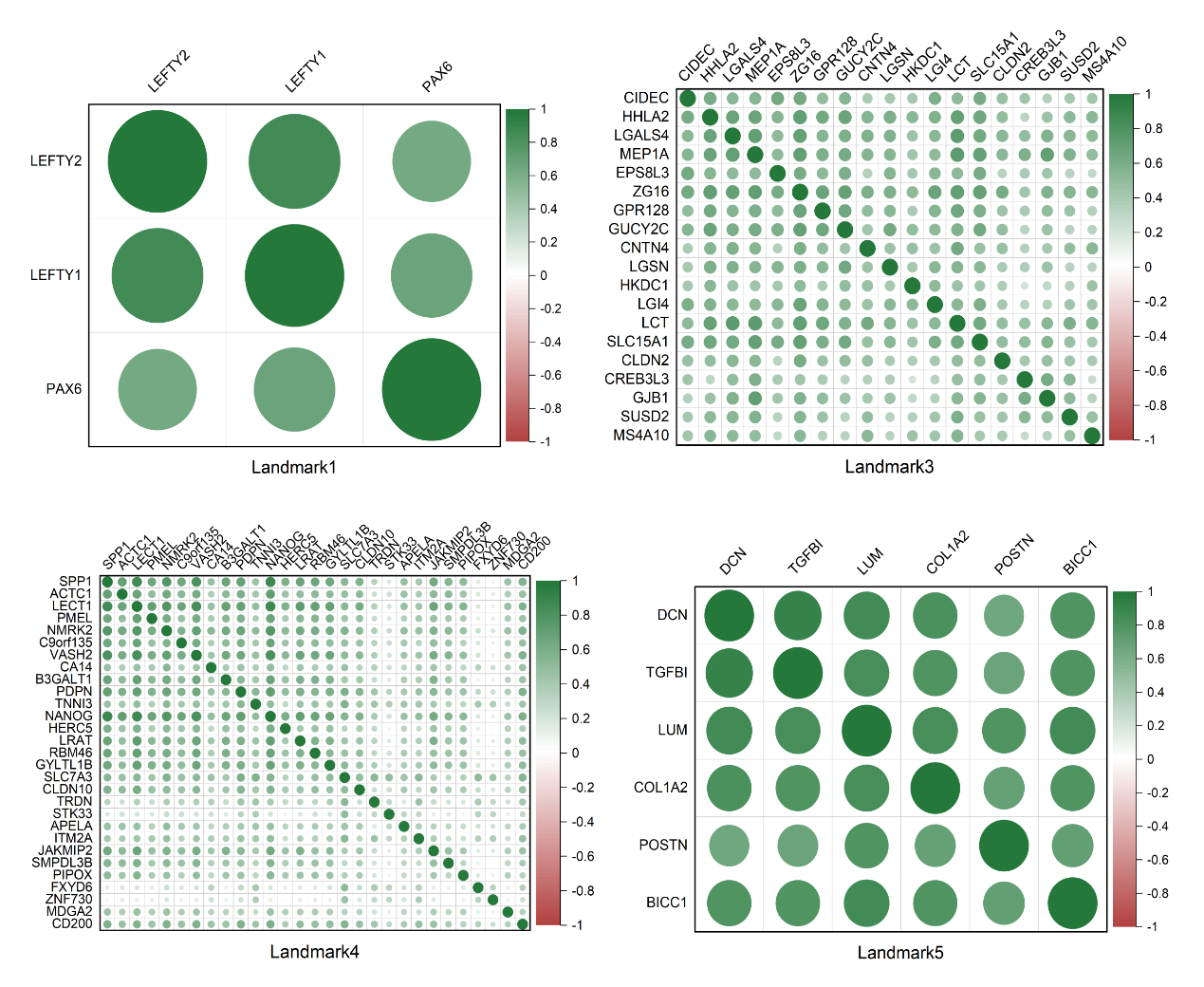


**Figure S5.** Dot plot of Pearson correlation coefficient of genes in four landmarks respectively identified by scQA on Camp2 datasets.


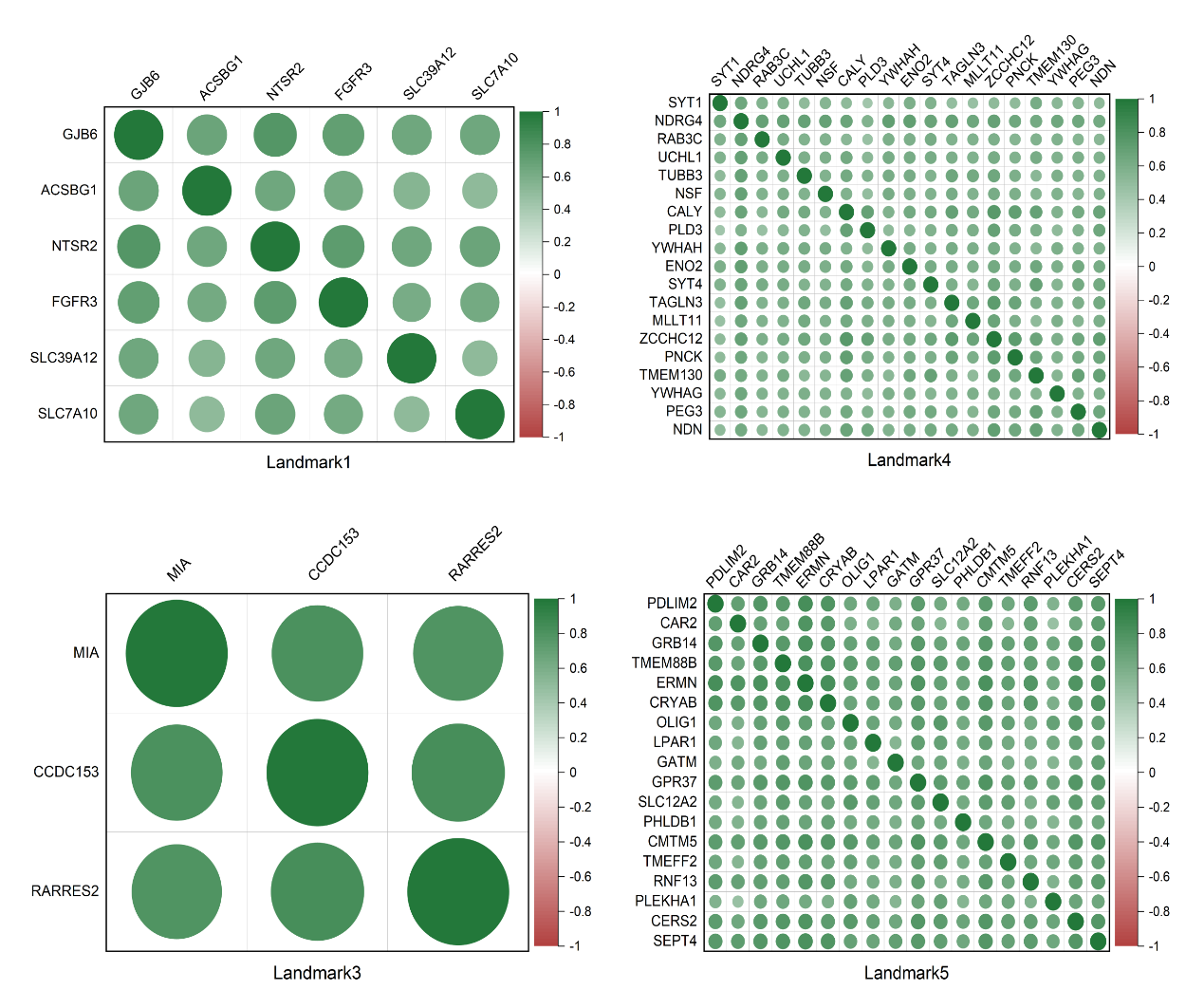


**Figure S6.** Dot plot of Pearson correlation coefficient of genes in four landmarks respectively identified by scQA on Romanov datasets.

**
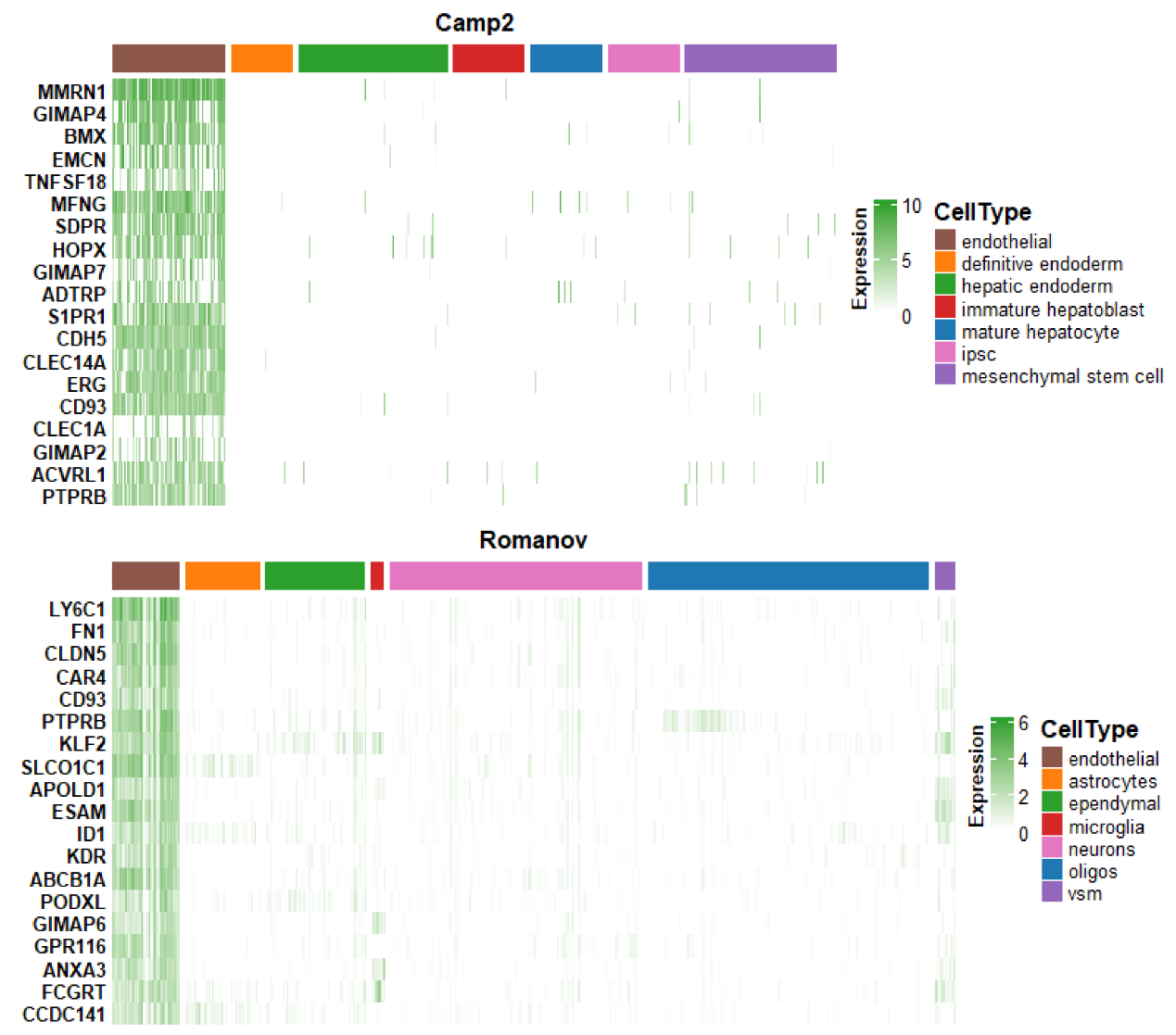
**

**Figure S7.** Heatmaps of genes in one landmark derived from Camp2 (top) and Romanov (bottom) datasets.


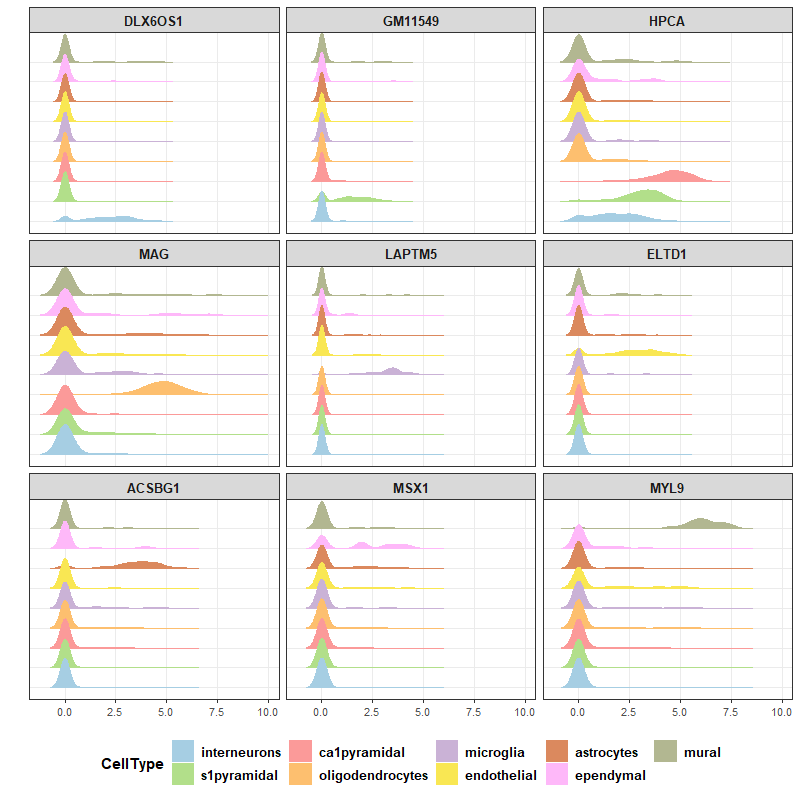


**Figure S8.** Joy plot of feature genes extracted by scQA on Zeisel dataset with landmark genes marked on the title of each subgraph, expression values represented on the x-axis and color representing the cell types.


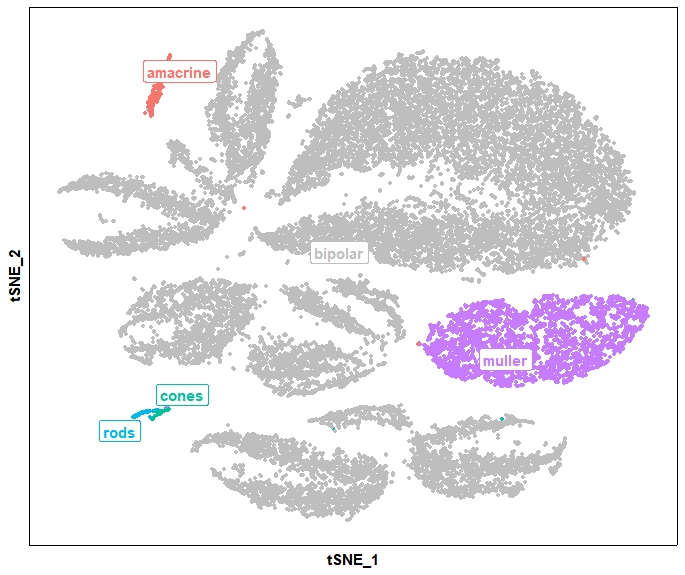


**Figure S9.** t-SNE visualization of five major cell types in Shekhar dataset.


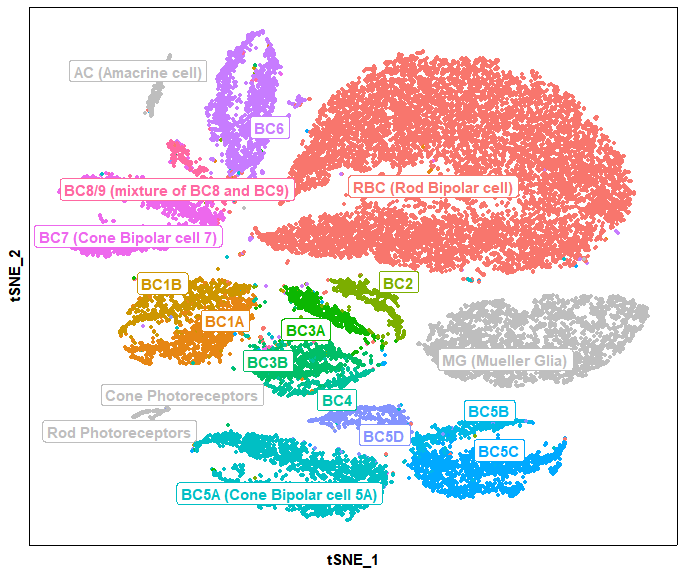


**Figure S10.** t-SNE visualization of bipolar cells in Shekhar dataset.


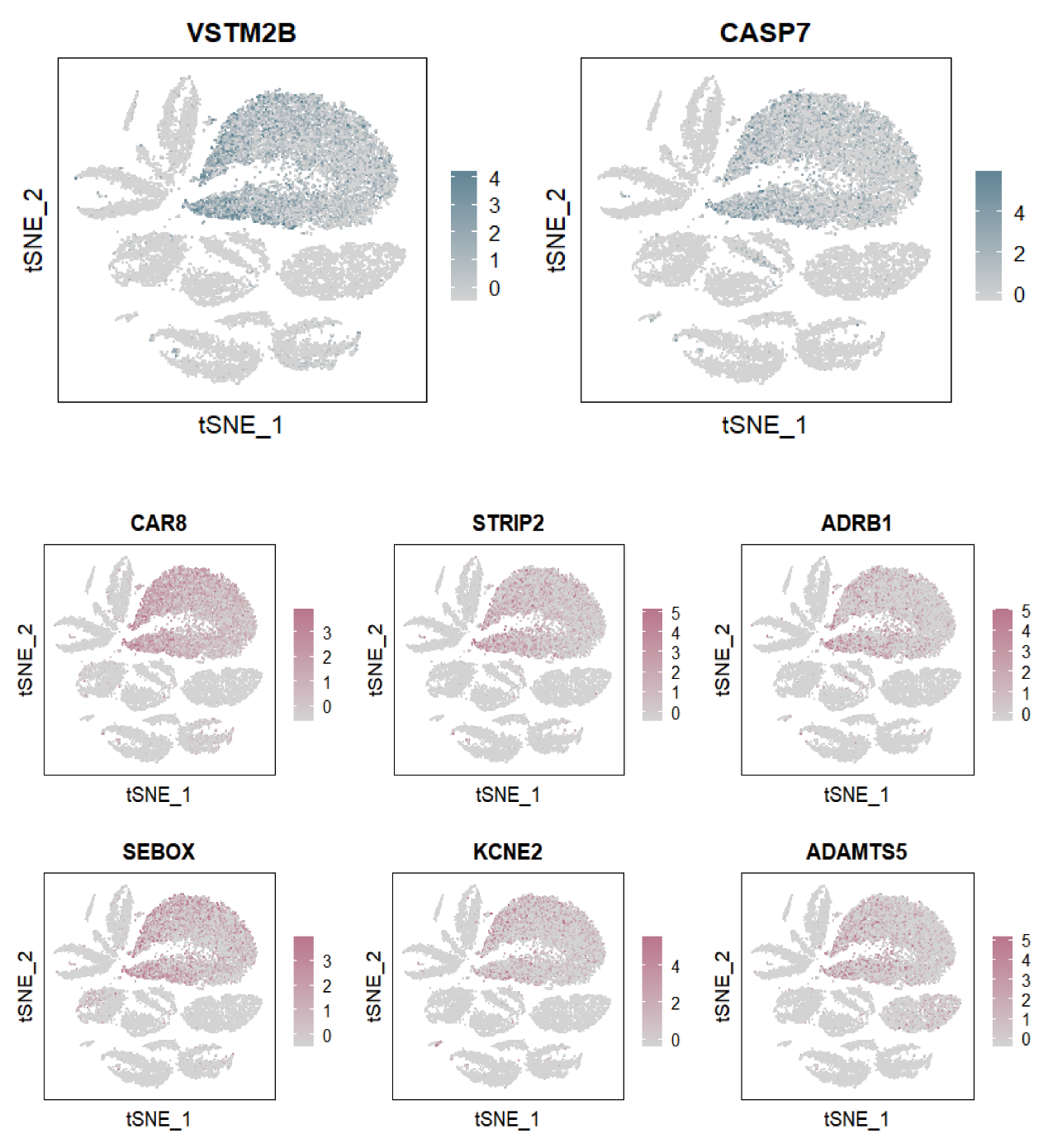


**Figure S11.** t-SNE visualizations of genes related to rod bipolar cell type in Shekhar dataset.


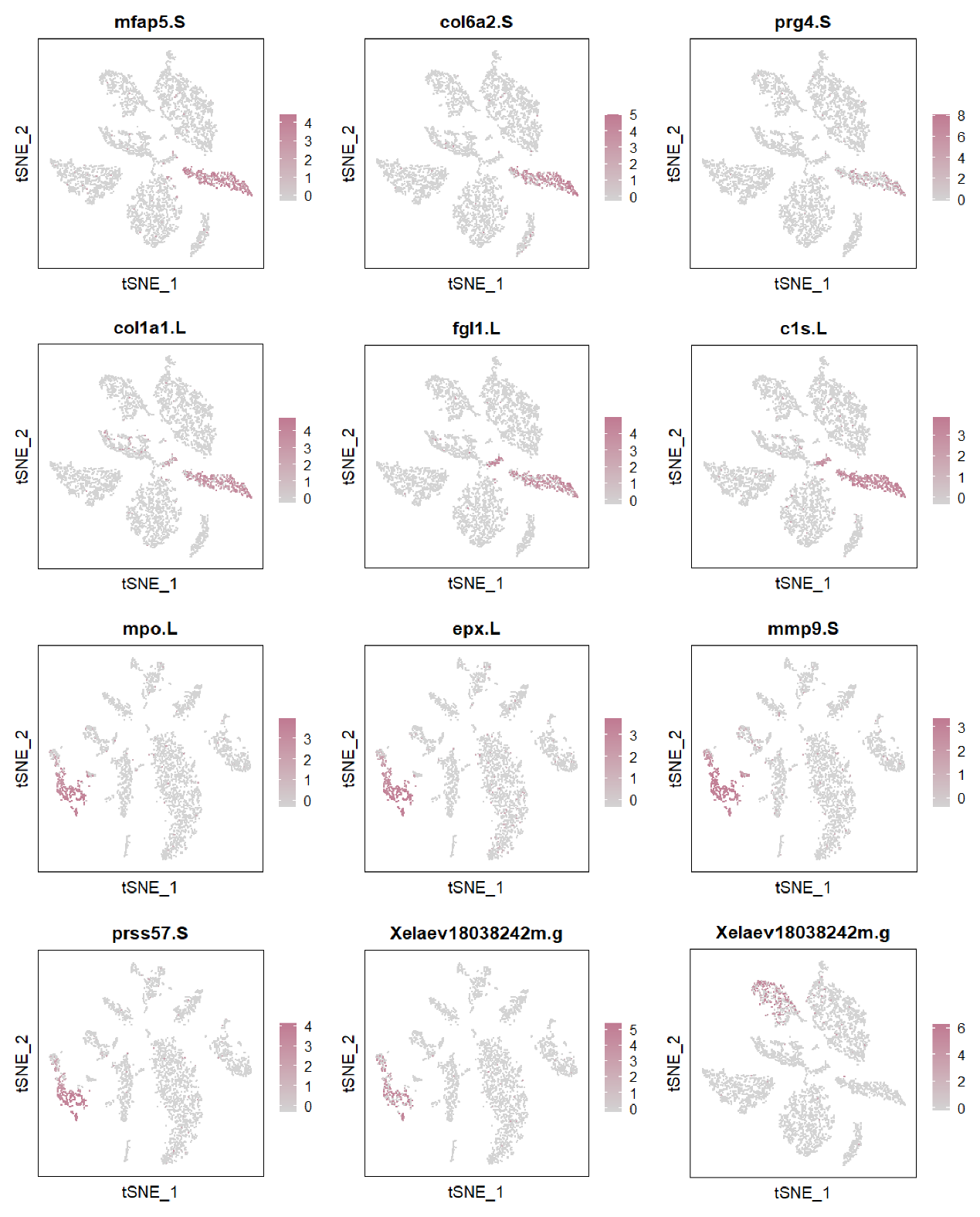


**Figure S12.** t-SNE visualizations of genes related to fibroblast in lung dataset and eosinophil in kidney dataset. The last one shows Xelaev18038242m.g in lung dataset.


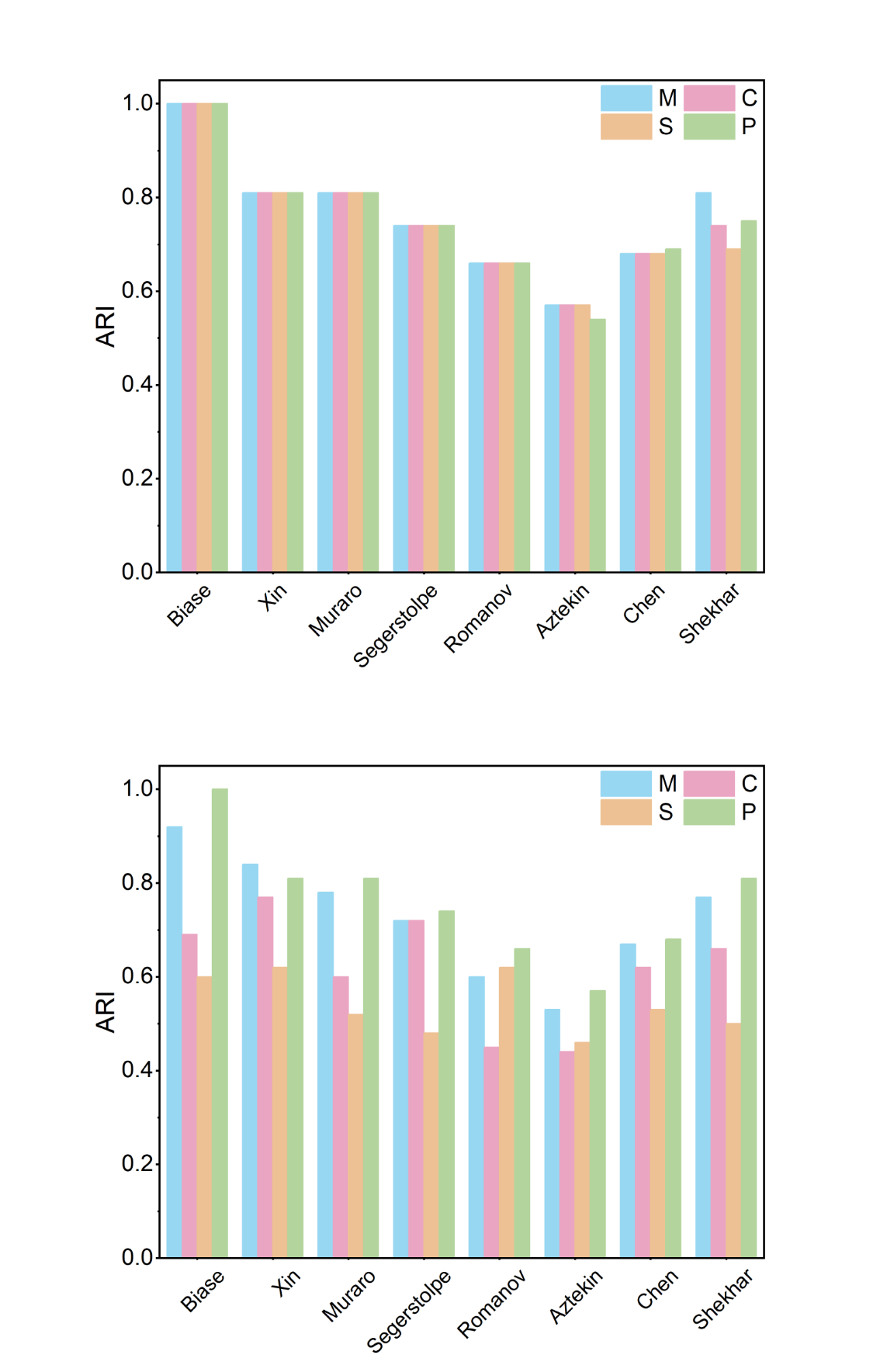


**Figure S13.** Bar plots of ARI scores with similarity calculations in the seed generation step using qualitative cell-landmark matrix $Q_{1}$ (top) and similarity calculations for constructing a directed nearest neighbor graph $G$ on quantitative cell-landmark matrix $Q_{2}$ (bottom) respectively. M, C, P, S stand for Manhattan distance, Chi-square score, Pearson correlation coefficient, Spearman's rank correlation coefficient respectively.
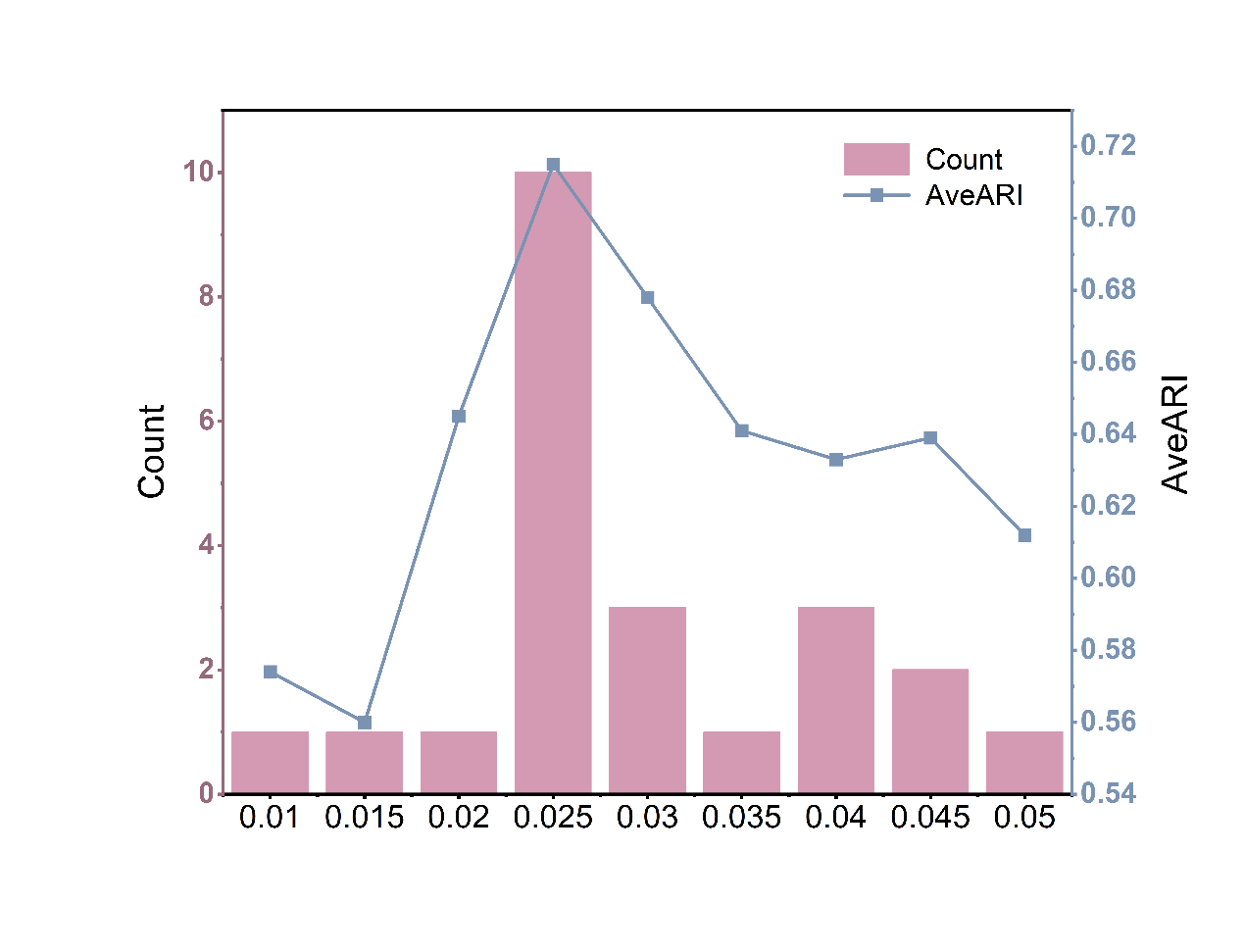


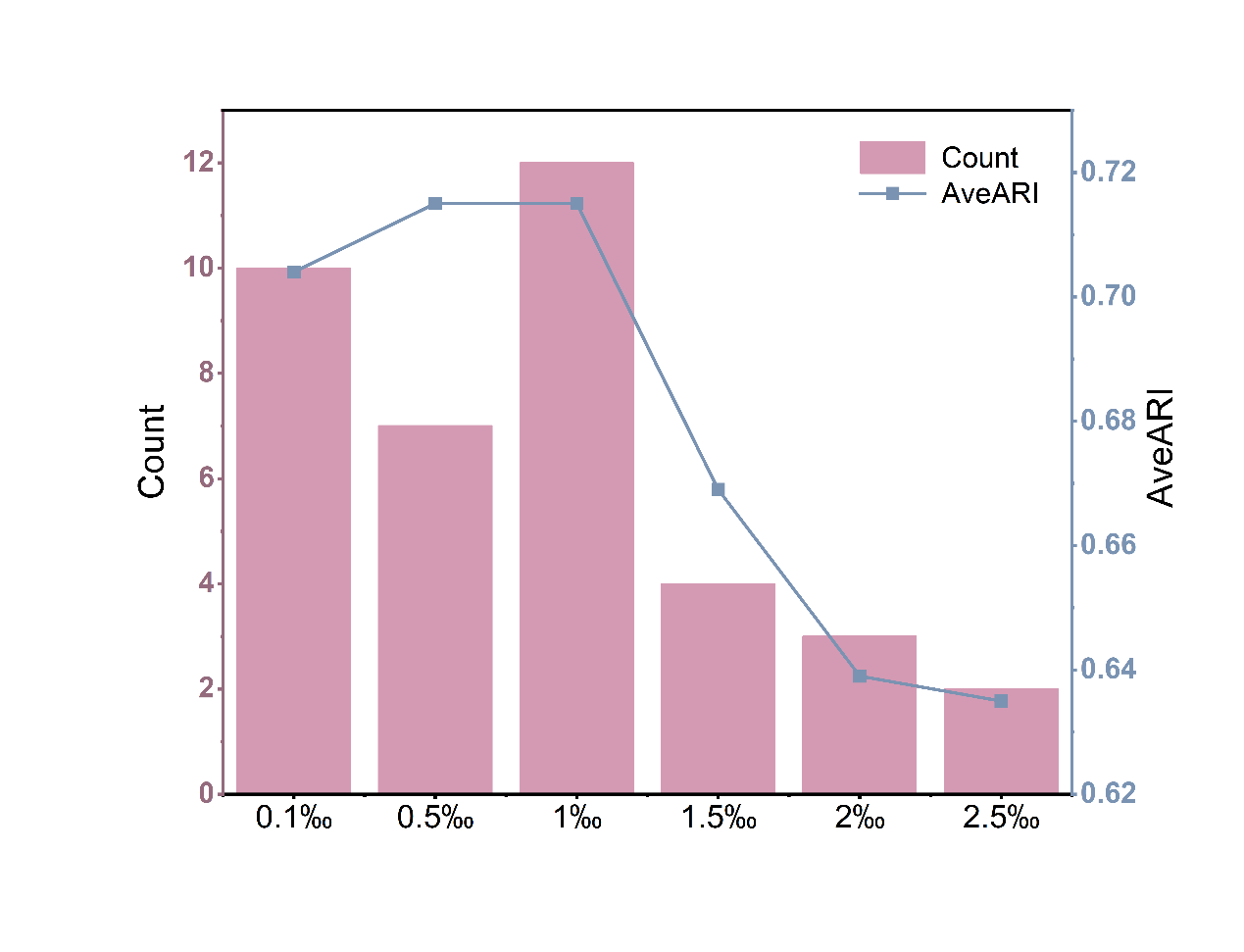


**Figure S14.** (top) Average ARI of 20 datasets with different thresholds of pre-processing. The filter thresholds range from 1% to 5% with step by 0.5%. (bottom) Average ARI of 20 datasets with different thresholds of retained gene pairs for constructing gene similarity graph. The percentage of gene pairs range from 0.1‰ to 2.5‰. Bar plots show the number of datasets with the highest ARI scores for the current parameter. Note that the highest ARI score for each dataset may appear in multiple parameters.


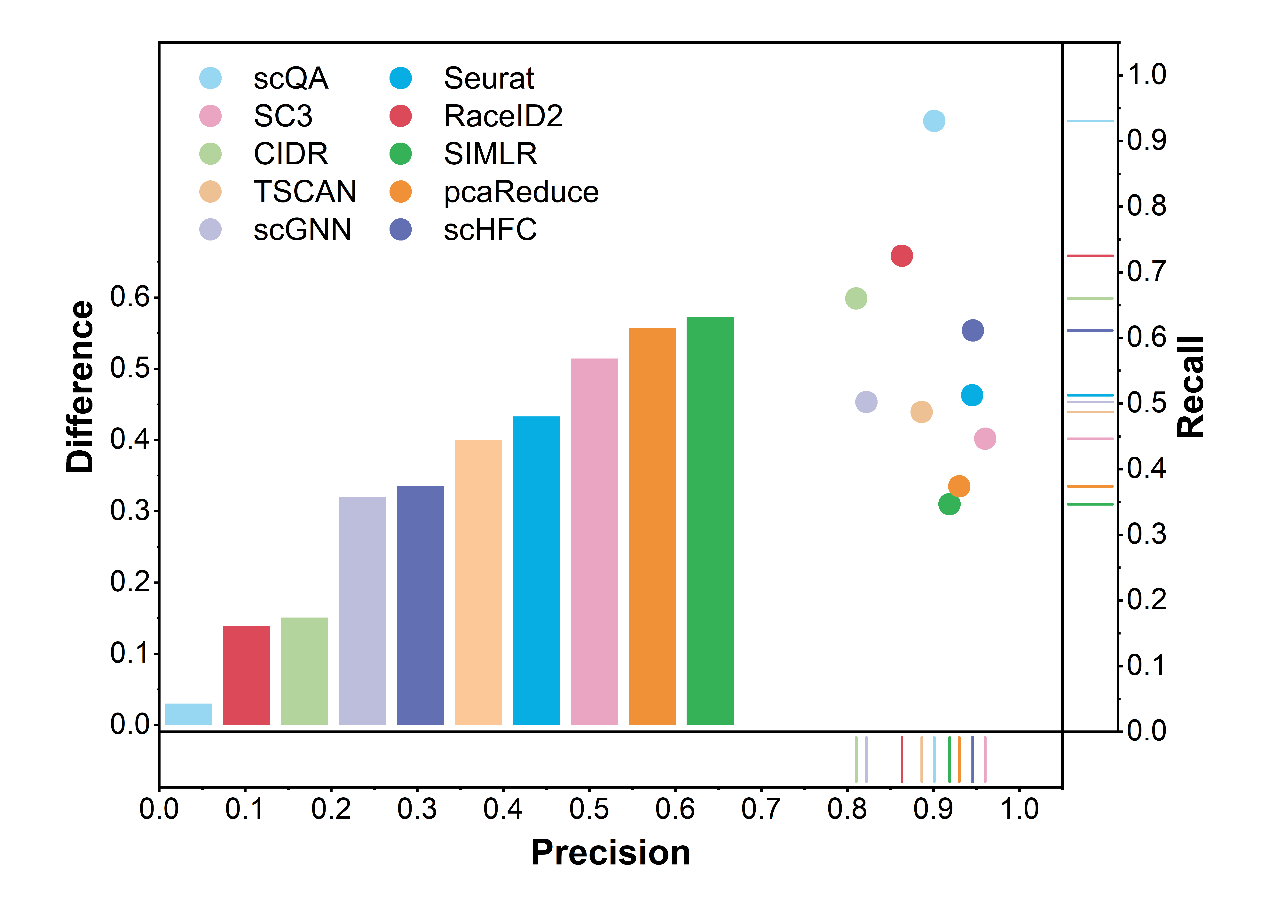


**Figure S15.** Average precision and recall scores of the largest cell types in the 20 datasets of 10 methods. Bar plots show the difference of precision and recall scores of the ten methods.


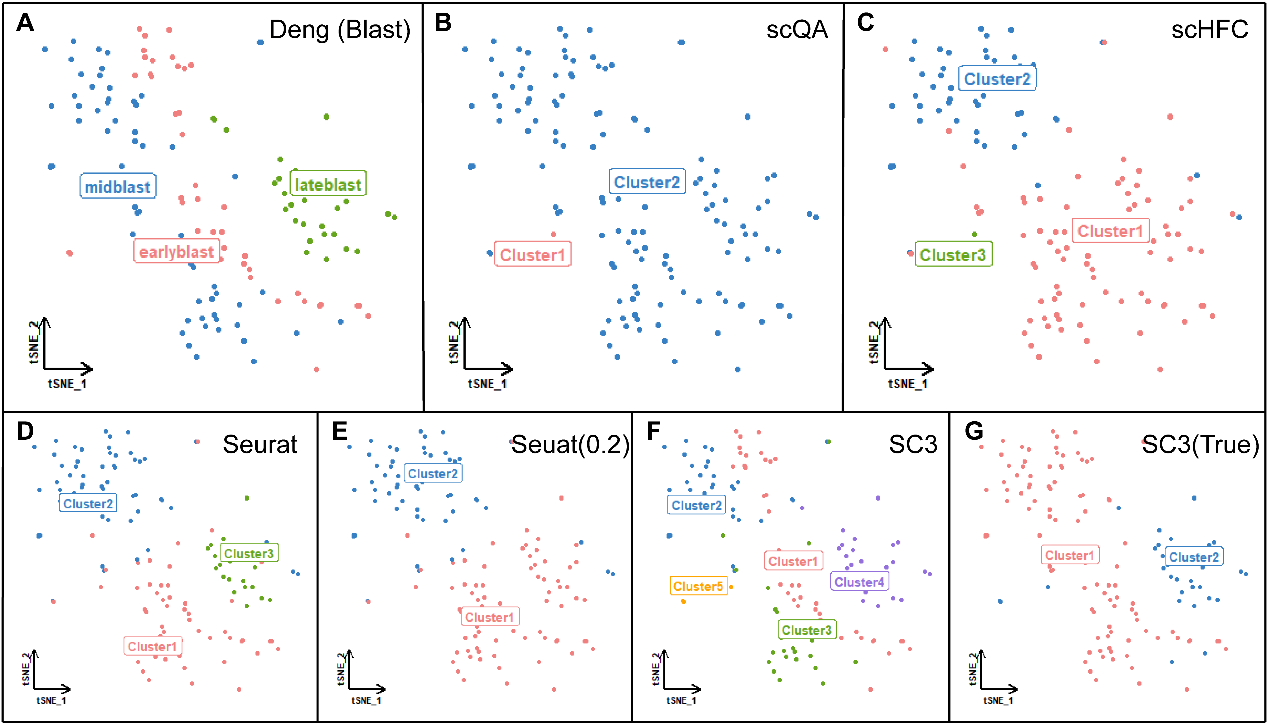


**Figure S16.** t-SNE visualizations of cells labeled Blast in Deng dataset. (A) True subtypes of blast cells. (B) Clusters of blast cells identified in scQA. (C) Clusters of blast cells identified in scHFC. (D-E) Clusters of blast cells identified in Seurat with resolution of default and 0.2 respectively. (F-G) Clusters of blast cells identified in SC3 with number of clusters identified by SC3 and true clusters.


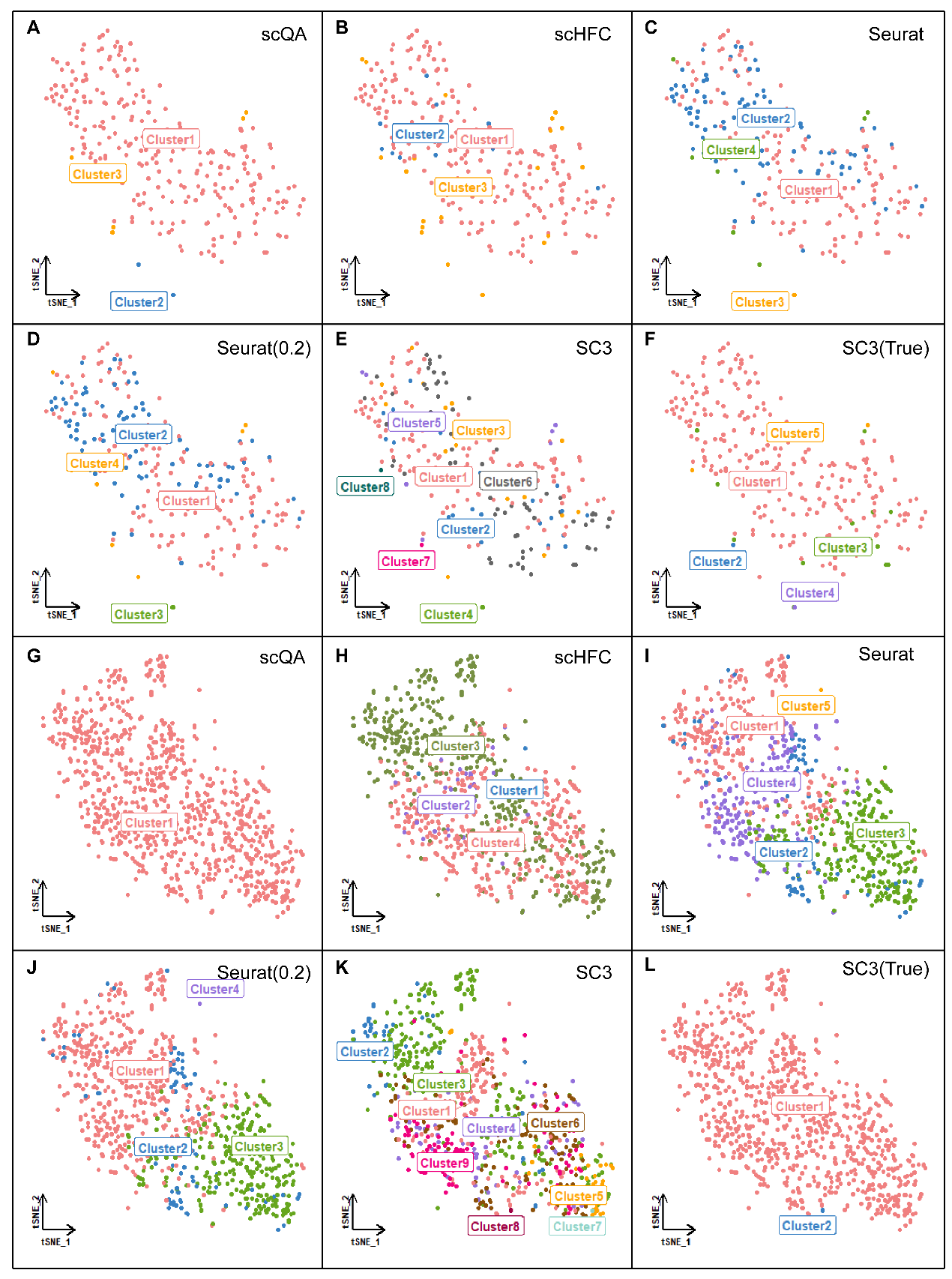


**Figure S17.** (A-F) t-SNE visualizations of Beta cells in Lawlor dataset clustered by scQA, scHFC, Seurat with default resolution, Seurat with resolution of 0.2, SC3 with number of clusters identified by SC3 and SC3 with number of clusters equal to true cell types. (G-L) t-SNE visualizations of Alpha cells in Xin dataset clustered by scQA, scHFC, Seurat with default resolution, Seurat with resolution of 0.2, SC3 with number of clusters identified by SC3 and SC3 with number of clusters equal to true cell types.


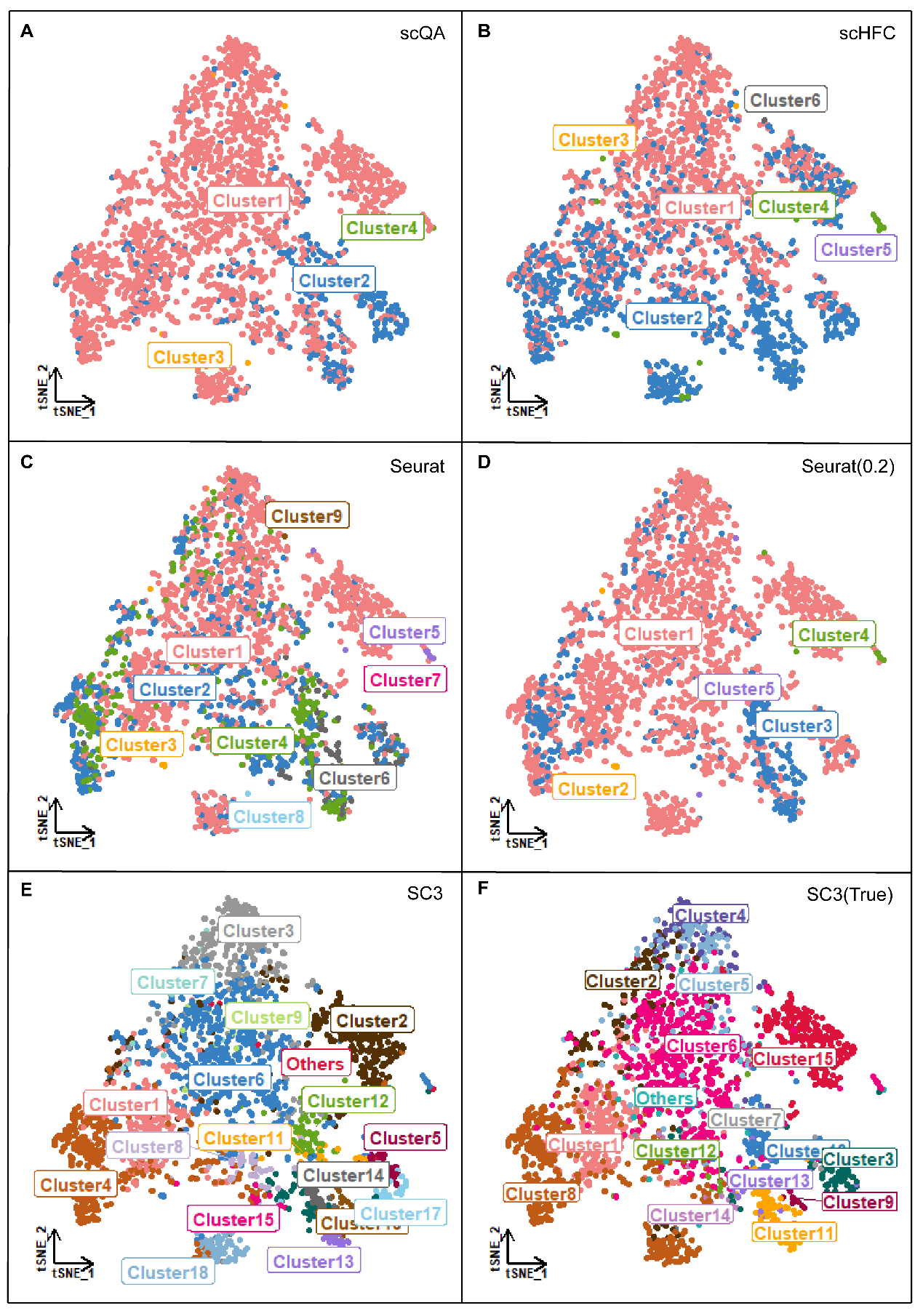


**Figure S18.** (A-F) t-SNE visualizations of Erythrocyte 1 cells in Aztekin dataset clustered by scQA, scHFC, Seurat with default resolution, Seurat with resolution of 0.2, SC3 with number of clusters identified by SC3 and SC3 with number of clusters equal to true cell types. For clearer visualization, in clustering results with more than ten clusters, clusters with fewer than 10 cells are merged into cluster ‘Others’.

# Supplementary Notes

**Note S1. Trend-preserved**

A term was used in biclustering papers. The expression patterns of two genes are said to be trend-preserving under certain conditions if and only if their expression vectors in the matrix are either order-preserving or order-reversing. Two vectors $x$ and $y$ are said to be order-preserving if and only if the corresponding elements in their respective vectors have the same rank (with respect to the numerical value), and order-reversing if and only if $x$ and $-y$ are order-preserving [10].

**Quasi-trend-preserved**

In this study, the term employed carries a slightly nuanced meaning, referring to genes that exhibit similar trends of expression, that is, the ranks defined by expression vectors are akin, although they might not be identical. The definition as follows:

Gene1 and Gene2 are quasi-trend-preserved in cells if and only if:

$$\begin{aligned} x_{i}\in\left( y_{i}-\delta,y_{i}+\delta\right), \forall i\in N\#\left( A.1 \right) \end{aligned}$$

Where $x$ and $y$ are expression vectors of Gene1 and Gene2 respectively. $N$ is the number of cells and $\delta\ll1$ is a small positive number.

For example:

|  | Cell1 | Cell2 | Cell3 | Cell4 | Cell5 |
| --- | --- | --- | --- | --- | --- |
| Gene1 | 0 | 0 | 0.1 | 0.2 | 0.8 |
| Gene2 | 0 | 0 | 0.2 | 0.1 | 0.9 |

These two genes are quasi-trend-preserving in all five cells according to our definition, despite their ranks are not the same. Each element in the expression vector of Gene2 is around the corresponding element in the expression vector of Gene1 within a certain range.

**Note S2. Complementary entropy**

Complementary entropy [11,12] is an index to measure the quality of clusters in a network. Let $C_{k}$ denotes cluster $k$, and $N_{i}\left( C_{k} \right)$ be the number of nodes in $C_{k}$ adjacent to node $v_{i}$. The fraction of nodes in $C_{k}$ adjacent to $v_{i}$ is denoted by $f_{i}\left( C_{k} \right)=\frac{N_{i}\left( C_{k} \right)}{\left| C_{k} \right|}$. $h_{i}=\sqrt{\sum_{k} \left( \frac{f_{i}\left( C_{k} \right)}{\sum_{r} f_{i}\left( C_{r} \right)} \right)^{2}}$ represents the degree to which node $v_{i}$ is separated from other clusters and $I_{i}=\sum_{k} N_{i}\left( C_{k} \right)\times f_{i}\left( C_{k} \right)$ evaluates how close the node $v_{i}$ is to other clusters. Then the complementary entropy is defined as

$$\begin{aligned} F=\sum_{i=1}^{n} h_{i}I_{i}\#\left( A.2 \right) \end{aligned}$$

**Note S3. Calculation of overlap odds**

Suppose there are three sets, $M1$, $M2$ and $M3$, the union of all sets is denoted as $N$, which equals to $\left| M1\cup M2\cup M3 \right|$. If the number of elements in an overlapped block in the Venn diagram is $n$, then the overlap odd is $\frac{n}{N}$. The intersection of $M1$ and $M2$ should be regarded as the sum of two blocks, $\left| M1\cap M2-M3 \right|$ and $\left| M1\cap M2\cap M3 \right|$. Therefore, the overlap odd of $M1$ and $M2$ is calculated as $\frac{\left| M1\cap M2-M3 \right|+\left| M1\cap M2\cap M3 \right|}{N}$.

**Note S4. Hypergeometric test**

**Hypergeometric distribution** is a discrete probability distribution that describes the probability of $k$ successes (random draws for which a successful draw means that the hub gene is drawn) in $n$ draws, without replacement, from a finite population of genes of size $N$ that contains exactly $M$ hub genes.

A random variable $x$ follows the hypergeometric distribution if its probability mass function is given by:

$$\begin{aligned} P\left( x=k \right)=\frac{\binom{M}{k}\binom{N-M}{n-k}}{\binom{N}{n}}\#\left( A.3 \right) \end{aligned}$$

**Hypergeometric test** uses the hypergeometric distribution to measure the statistical significance of having drawn a sample consisting of a specific number of $k$ hub genes (out of $n$ total draws) from a gene set of size $N$ containing $M$ hub genes. The hypergeometric p-value is calculated as the probability of randomly drawing $k$ or more hub genes from the population in $n$ total draws.

**Note S5. Similarity metrics**

**Manhattan distance** between two gene vectors $\boldsymbol{u}=\left( u_{1},u_{2},\cdots,u_{n} \right)$ and $\boldsymbol{v}=\left( v_{1},v_{2},\cdots,v_{n} \right)$ is calculated as:

$$\begin{aligned} \boldsymbol{M}\left( \boldsymbol{u}\mathbf{,}\boldsymbol{v} \right)\boldsymbol{=}\left\| \boldsymbol{u-v} \right\|\mathbf{=}\sum_{\boldsymbol{i=1}}^{\boldsymbol{n}} \left| u_{i}\boldsymbol{-}v_{i} \right|\boldsymbol{\#}\left( A.4 \right) \end{aligned}$$

And the similarity between $\boldsymbol{u}$ and $\boldsymbol{v}$ is $1-\frac{\boldsymbol{M}\left( \boldsymbol{u}\mathbf{,}\boldsymbol{v} \right)}{n}$.

**Chi-square score** between two gene vectors $\boldsymbol{u}$ and $\boldsymbol{v}$ is defined as $\frac{sign\left( AD-BC \right)\left( AD-BC \right)^{2}}{\left( A+B \right)\left( A+C \right)\left( B+D \right)\left( C+D \right)}$. Where $A$ is the number of cells in which both $\boldsymbol{u}$ and $\boldsymbol{v}$ are expressed, $B$ is the number of cells in which $\boldsymbol{u}$ is expressed but $\boldsymbol{v}$ is not, $C$ is the numbers of cells where $\boldsymbol{v}$ is expressed but $\boldsymbol{u}$ is not, and $D$ is the number of cells where both $\boldsymbol{u}$ and $\boldsymbol{v}$ are unexpressed [13].

**Pearson correlation coefficient** between two gene vectors $\boldsymbol{u}$ and $\boldsymbol{v}$ is formulated as $\boldsymbol{\rho}\mathbf{=}\frac{\boldsymbol{cov}\left( \boldsymbol{u,v} \right)}{\boldsymbol{\sigma}_{\boldsymbol{u}}\boldsymbol{\sigma}_{\boldsymbol{v}}}$. Where $\boldsymbol{cov}\left( \boldsymbol{u}\mathbf{,}\boldsymbol{v} \right)\boldsymbol{=E}\left[ \left( \boldsymbol{u-}\boldsymbol{\mu}_{\boldsymbol{u}} \right)\left( \boldsymbol{v-}\boldsymbol{\mu}_{\boldsymbol{v}} \right) \right]$**,** $\boldsymbol{\mu}$ is the mean and $\boldsymbol{\sigma}$ is the standard deviation.

**Spearman's rank correlation coefficient** between two gene vectors $\boldsymbol{u}$ and $\boldsymbol{v}$ is formulated as $\boldsymbol{r}\mathbf{=}\frac{\boldsymbol{cov}\left( \boldsymbol{R}\left( \boldsymbol{u} \right)\boldsymbol{,R}\left( \boldsymbol{v} \right) \right)}{\boldsymbol{\sigma}_{\boldsymbol{R}\left( \boldsymbol{u} \right)}\boldsymbol{\sigma}_{\boldsymbol{R}\left( \boldsymbol{v} \right)}}$. Where $\boldsymbol{R}\left( \boldsymbol{u} \right)$ and $\boldsymbol{R}\left( \boldsymbol{v} \right)$ are rank variables of $\boldsymbol{u}$ and $\boldsymbol{v}$ respectively.

**Note S6. Comparison with other tools**

SC3 is a consensus method developed by performing *k*-means on multiple matrices. Seurat is developed by performing PCA followed by recalculating the similarities based on shared nearest neighbors. CIDR is an ultrafast method developed by performing PCA based on zero-imputed similarities. Like SC3, pcaReduce is an agglomerative clustering approach developed by combining PCA and *k*-means. SIMLR is developed by learning a similarity matrix via combining multiple kernels. TSCAN is designed by combining PCA with model-based clustering method. RaceID2 is designed based on Pearson correlation coefficient to identify rare and abundant cell types using *k*-medoids. scGNN is a deep learning-based method and integrates graph convolutional network (GCN) into multi-autoencoder to improve clustering results. Based on Fuzzy C Mean (FCM) and Gath-Geva algorithms, scHFC performs a hybrid fuzzy clustering. For SC3 method, a hybrid approach combining SVM was utilized when cells in the dataset exceeded 5,000. For datasets containing over 10,000 cells, large scale SIMLR was used as a substitution. scGNN utilized LTMG for its execution. The range of estimation of *k* was set to 2 to 50 if it was needed to be specified. As for the remaining parameters utilized in these methods, their default values were employed for the comparisons within this paper. All experiments were carried out on a Linux server.

**Note S7. Internal evaluation metrics**

**Silhouette Coefficient**[14]

For data point $i\in C_{I}$ ($\left| C_{I} \right|>1$), the mean distance between $i$ and all other data in $C_{I}$ is defined as:$a\left( i \right)=\frac{1}{\left| C_{I} \right|-1}\sum_{j\in C_{I}, j\neq i} d(i,j)$, where $\left| C_{I} \right|$ is the number of points belonging to $C_{J}$, and $d(i,j)$ is the distance between $i$ and $j$. The mean distance between $i$ and $C_{J}$ (where $C_{J}\neq C_{I}$) is defined as the mean distance from $i$ to all data point in $C_{J}$. We now define $b\left( i \right)=\min_{J\neq I} \frac{1}{\left| C_{J} \right|}\sum_{j\in C_{J}} d(i,j)$. Then Silhouette value of $i$ is defined as

$$\begin{aligned} s\left( i \right)=\frac{b\left( i \right)-a\left( i \right)}{\max\left\{ a\left( i \right), b\left( i \right) \right\}}\#\left( A.5 \right) \end{aligned}$$

**Calinski–Harabasz index**[15]

Calinski–Harabasz Index is defined as the ratio of the between-cluster separation to the within-cluster dispersion, normalized by their number of degrees of freedom:

$$\begin{aligned} CH=\frac{\frac{BC}{k-1}}{\frac{WC}{k-1}}\#\left( A.6 \right) \end{aligned}$$

where $k$ is the number of clusters, $n$ is the number of data points.

The between-cluster separation is calculated as $BC=\sum_{i=1}^{k} n_{i}\left\| c_{i}-c \right\|^{2}$, where $n_{i}$ is the number of points in $C_{I}$, $c_{i}$ is the centroid of $C_{I}$ and $c$ is the overall centroid of all data.

The within-cluster dispersion is calculated as $WC=\sum_{i=1}^{k} \sum_{j\in C_{J}} \left\| j-c_{i} \right\|^{2}$.

**Davies–Bouldin index**[16]

Let $R_{ij}$ be a measure of how good the clustering scheme is defined as $R_{ij}=\frac{S_{i}+S_{j}}{M_{ij}}$, where $S_{i}=\left( \frac{1}{n_{i}}\sum_{j=1}^{n_{i}} \left\| X_{j}-c_{i} \right\|_{p}^{q} \right)^{\frac{1}{q}}$, $X_{j}$ is a feature vector, $c_{i}$ is the centroid of $C_{I}$ and $n_{i}$ is the number of points in $C_{I}$. $M_{ij}$ is defined as: $M_{ij}=\left\| c_{i}-c_{j} \right\|_{p}$,which measures the seperation between $C_{I}$ and $C_{J}$.

Davies–Bouldin index is calculated as:

$$\begin{aligned} DB=\frac{1}{k}\sum_{i=1}^{k} \max_{j\neq i} R_{ij}\#\left( A.6 \right) \end{aligned}$$

where $k$ is the number of clusters.

**Note S8. Clustering analysis for large major cell types**

To offer a comprehensive comparison of the clustering results from diverse methods, we calculated the recall and precision for the largest cluster in each dataset. The results are depicted in the Fig. S15. We observed that there was a negligible variance in precision among different methods, but a substantial discrepancy was evident in recall. This discrepancy in recall might stem from the identification of subtypes within the major cell type. We singled out three methods scHFC, Seurat and SC3 that exhibited higher accuracy but lower recall for subsequent analysis together with scQA. In our examination of the Deng dataset, we opted to visualize the largest cluster, named “blast” (Fig. S16). Comparing the clustering results of blast cells from the four methods with the true subtypes within the blast cell type, we found that while both scHFC and Seurat correctly identified total number of subtypes, they failed to accurately discern the specific subtypes. Seurat correctly pinpointed the lateblasts, SC3 recognized midblasts and lateblasts, whereas scHFC identified two incorrect subtypes and outliers (Cluster3). Similarly, scQA pinpointed this outlier as Cluster1. Adjusting Seurat's resolution (by a step size of 0.2 to cluster at a number equal to or less than the true cell types) and providing SC3 with the true cell types still led to inaccuracies in their identification of subtypes within the major cell type. SC3 identified one subtype out of the three, while Seurat mistakenly identified two subtypes but both of them failed to capture the entirety of the major cell type. Additionally, with the number of clusters set to be less than or equal to the real cell types, both SC3 and Seurat tended to merge other cell types into one cluster, indicating a focus on differences among subtypes within the large major cell type, leading to the erroneously merging of the other two smaller major cell types.

In the Lawlor dataset, scQA accurately identified the beta cell type, while scHFC misallocated some cells to Cluster2, a similar situation was observed in Seurat (Fig. S17). We lowered Seurat's resolution to 0.2, causing the identified clusters to be fewer than the actual cell types, yet Seurat still couldn't correctly identify the entire cell type. Visual analysis indicated that SC3 fragmented beta cells into unreliable subtypes, but identified the entirety of beta cell type only after providing SC3 with the true cell type number. The same occurred in alpha cells in the Xin dataset (Fig. S17). For Seurat, reducing the resolution led to the integrality of alpha cells, albeit at the expense of clustering other cell types into one cluster. When equipped with the true cell type number, SC3 could identify this major cell type. Lastly, in analyzing the Erythrocyte 1 cells in the Aztekin dataset (Fig. S18), even when providing SC3 with the correct cell type number, it still subdivided Erythrocyte 1 cells into multiple subclusters instead of correctly distinguishing different major cell types. Overall, scHFC and scQA, using default parameters, were able to identify major cell types more comprehensively, seemingly emphasizing differences between major cell types. Conversely, SC3 and Seurat tended to focus on potential divisions within larger cell types, resulting in inaccurate subclusters. Moreover, even with prior information, these methods tended to prioritize internal differences within larger cell types rather than accurately identifying discrepancies between different major cell types.

# References

[1] V.Y. Kiselev, K. Kirschner, M.T. Schaub, T. Andrews, A. Yiu, T. Chandra, K.N. Natarajan, W. Reik, M. Barahona, A.R. Green, M. Hemberg, SC3: consensus clustering of single-cell RNA-seq data, Nat Methods. 14 (2017) 483-+. https://doi.org/10.1038/nmeth.4236.

[2] R. Satija, J.A. Farrell, D. Gennert, A.F. Schier, A. Regev, Spatial reconstruction of single-cell gene expression data, Nat Biotechnol. 33 (2015) 495-U206. https://doi.org/10.1038/nbt.3192.

[3] P.J. Lin, M. Troup, J.W.K. Ho, CIDR: ultrafast and accurate clustering through imputation for single-cell RNA-seq data, Genome Biol. 18 (2017) 59. https://doi.org/10.1186/s13059-017-1188-0.

[4] J. Zurauskiene, C. Yau, pcaReduce: hierarchical clustering of single cell transcriptional profiles, BMC Bioinformatics. 17 (2016) 140. https://doi.org/10.1186/s12859-016-0984-y.

[5] B. Wang, J.J. Zhu, E. Pierson, D. Ramazzotti, S. Batzoglou, Visualization and analysis of single-cell RNA-seq data by kernel-based similarity learning, Nat Methods. 14 (2017) 414-+. https://doi.org/10.1038/nmeth.4207.

[6] Z.C. Ji, H.K. Ji, TSCAN: pseudo-time reconstruction and evaluation in single-cell RNA-seq analysis, Nucleic Acids Res. 44 (2016) e117. https://doi.org/10.1093/nar/gkw430.

[7] D. Grun, M.J. Muraro, J.C. Boisset, K. Wiebrands, A. Lyubimova, G. Dharmadhikari, M. van den Born, J. van Es, E. Jansen, H. Clevers, E.J.P. de Koning, A. van Oudenaarden, De Novo prediction of stem cell identity using single-cell transcriptome data, Cell Stem Cell. 19 (2016) 266–277. https://doi.org/10.1016/j.stem.2016.05.010.

[8] J.X. Wang, A.J. Ma, Y.Z. Chang, J.T. Gong, Y.X. Jiang, R. Qi, C.K. Wang, H.J. Fu, Q. Ma, D. Xu, scGNN is a novel graph neural network framework for single-cell RNA-Seq analyses, Nat Commun. 12 (2021) 1882. https://doi.org/10.1038/s41467-021-22197-x.

[9] J. Wang, J.F. Xia, D.Y. Tan, R.X. Lin, Y. Su, C.H. Zheng, scHFC: a hybrid fuzzy clustering method for single-cell RNA-seq data optimized by natural computation, Brief Bioinform. 23 (2022) bbab588. https://doi.org/10.1093/bib/bbab588.

[10] X.Y. Liu, D. Li, J.T. Liu, Z.C. Su, G.J. Li, RecBic: a fast and accurate algorithm recognizing trend-preserving biclusters, Bioinformatics. 36 (2020) 5054–5060. https://doi.org/10.1093/bioinformatics/btaa630.

[11] L. Bai, X.Q. Cheng, J.Y. Liang, Y.K. Guo, Fast graph clustering with a new description model for community detection, Inf Sci (N Y). 388 (2017) 37–47. https://doi.org/10.1016/j.ins.2017.01.026.

[12] G. Yang, W.P. Zheng, C.H. Che, W.J. Wang, Graph-based label propagation algorithm for community detection, International Journal of Machine Learning and Cybernetics. 11 (2020) 1319–1329. https://doi.org/10.1007/s13042-019-01042-0.

[13] P. Qiu, Embracing the dropouts in single-cell RNA-seq analysis, Nat Commun. 11 (2020) 1169. https://doi.org/10.1038/s41467-020-14976-9.

[14] P.J. Rousseeuw, Silhouettes: A graphical aid to the interpretation and validation of cluster analysis, J Comput Appl Math. 20 (1987) 53–65. https://doi.org/https://doi.org/10.1016/0377-0427(87)90125-7.

[15] T. Caliński, J. Harabasz, A dendrite method for cluster analysis, Communications in Statistics. 3 (1974) 1–27. https://doi.org/10.1080/03610927408827101.

[16] D.L. Davies, D.W. Bouldin, A Cluster Separation Measure, IEEE Trans Pattern Anal Mach Intell. PAMI-1 (1979) 224–227. https://doi.org/10.1109/TPAMI.1979.4766909.
